# Comparative Epigenomics Reveals that RNA Polymerase II Pausing and Chromatin Domain Organization Control Nematode piRNA Biogenesis

**DOI:** 10.1016/j.devcel.2018.12.026

**Published:** 2019-03-25

**Authors:** Toni Beltran, Consuelo Barroso, Timothy Y. Birkle, Lewis Stevens, Hillel T. Schwartz, Paul W. Sternberg, Hélène Fradin, Kristin Gunsalus, Fabio Piano, Garima Sharma, Chiara Cerrato, Julie Ahringer, Enrique Martínez-Pérez, Mark Blaxter, Peter Sarkies

**Affiliations:** 1 MRC London Institute of Medical Sciences, London W12 0NN, UK; 2 Institute of Clinical Sciences, Imperial College London, London W12 0NN, UK; 3 Institute of Evolutionary Biology, School of Biological Sciences, University of Edinburgh, Edinburgh EH9 3TF, UK; 4 Division of Biology and Biological Engineering, California Institute of Technology, Pasadena, CA 91125, USA; 5 Department of Biology, New York University, New York, NY 10003, USA; 6 Center for Genomics and Systems Biology, New York University, New York, NY 10003, USA; 7 Center for Genomics and Systems Biology, New York University Abu Dhabi, Abu Dhabi, United Arab Emirates; 8 The Gurdon Institute and Department of Genetics, University of Cambridge, Cambridge, UK

**Keywords:** piwi-interacting small RNAs, *C. elegans*, evolution, chromatin, epigenetics, nematodes, comparative epigenomics

## Abstract

Piwi-interacting RNAs (piRNAs) are important for genome regulation across metazoans, but their biogenesis evolves rapidly. In *Caenorhabditis elegans*, piRNA loci are clustered within two 3-Mb regions on chromosome IV. Each piRNA locus possesses an upstream motif that recruits RNA polymerase II to produce an ∼28 nt primary transcript. We used comparative epigenomics across nematodes to gain insight into the origin, evolution, and mechanism of nematode piRNA biogenesis. We show that the piRNA upstream motif is derived from core promoter elements controlling snRNA transcription. We describe two alternative modes of piRNA organization in nematodes: in *C. elegans* and closely related nematodes, piRNAs are clustered within repressive H3K27me3 chromatin, while in other species, typified by *Pristionchus pacificus*, piRNAs are found within introns of active genes. Additionally, we discover that piRNA production depends on sequence signals associated with RNA polymerase II pausing. We show that pausing signals synergize with chromatin to control piRNA transcription.

## Highlights

• Nematode piRNA transcription evolved from small nuclear RNA biogenesis
• Clustered piRNAs are produced from regulated (H3K27me3) chromatin domains
• Dispersed piRNAs are produced from active (H3K36me3) chromatin domains
• RNA polymerase II pausing determines the short (∼28 nt) length of piRNA precursors

## Graphical Abstract

**Figure:**
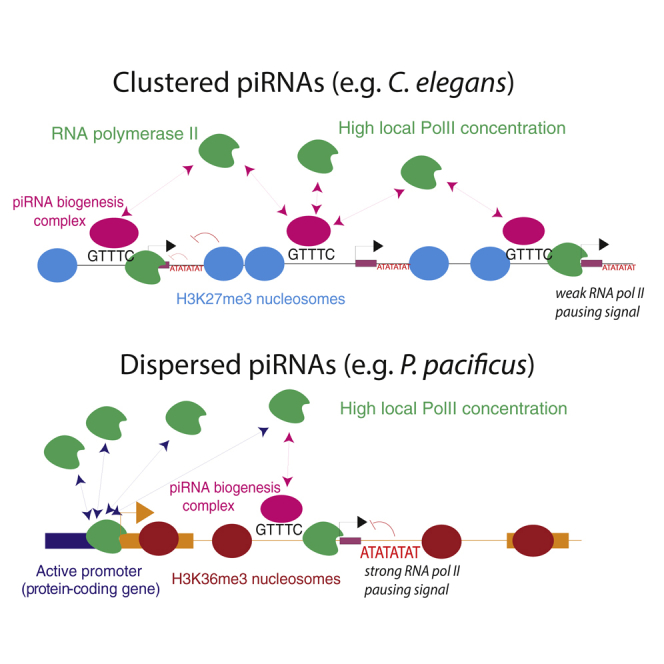

## Teaser

piRNAs are an important genome regulatory mechanism conserved across metazoans. In the nematode *C. elegans*, piRNA biogenesis evolved several differences from other metazoans. Beltran et al. study the origin of these differences through an evolutionary approach, discovering that chromatin environment and RNA polymerase II pausing synergize in piRNA biogenesis.

## Introduction

piRNAs are 21–30 nucleotide (nt) small RNAs that bind to members of the Piwi subfamily of Argonaute proteins. Conserved across animals, their ancestral role appears to be to defend the genome against transposable elements (TEs). piRNAs hybridize to TE-derived RNAs, instigating post-transcriptional and transcriptional silencing of TEs (Siomi et al., 2011). In many organisms, piRNAs are essential for fertility, and germ cell development is defective in their absence (Weick and Miska, 2014). Fertility defects in mutants lacking piRNAs can occur in the absence of defective TE silencing (Gou et al., 2014, Simon et al., 2014, Vourekas et al., 2012); thus, piRNAs are likely to have further, poorly understood, functions in germline development.

piRNA biogenesis has been characterized in arthropods and mammals. In these taxa, long precursor RNAs are produced from ∼100 genomic loci and processed into mature 26- to 30-nt piRNAs bound to Piwi proteins. In germ cells, primary piRNA processing occurs as part of a ping-pong cycle that amplifies piRNAs targeted to expressed TEs (Brennecke et al., 2007).

Despite the conservation of the general logic of piRNA pathway function and biogenesis across animals, the piRNA machinery diverges rapidly among closely related organisms. Many of the dedicated piRNA biogenesis factors characterized in *Drosophila melanogaster* are not conserved even among Diptera, let alone across arthropods (Weick and Miska, 2014). Some aspects of piRNA function are idiosyncratic. For example, in the mosquito *Aedes aegypti*, piRNAs derived from RNA viruses are found in the gut (Miesen et al., 2015), while in the silkworm *Bombyx mori*, piRNAs regulate a specific protein-coding target gene in the sex determination pathway (Kiuchi et al., 2014). How and why piRNA function and biogenesis diverges so rapidly remains unclear.

Nematodes (phylum Nematoda) represent an extreme example of the diversity of the piRNA system. Although piRNAs are conserved within the Rhabditina (Clade V of Blaxter et al., 1998), the entire piRNA pathway has been lost independently in several lineages across the phylum (Sarkies et al., 2015). Rhabditina contains the model nematode *Caenorhabditis elegans*, in which characterization of piRNAs is most advanced. As in other metazoans, piRNAs in *C. elegans* (termed 21U, for their typical length and 5′ uracil) associate with a Piwi protein, PRG-1, target TEs, and are important for fertility (Batista et al., 2008, Das et al., 2008). However, the *C. elegans* piRNA system displays intriguing differences to other organisms. First, the ping-pong cycle is not present. Instead, piRNAs recruit RNA-dependent RNA polymerases (RdRPs) to target RNAs. RdRP activity produces 22-nt RNAs that have a 5′ G (22G RNAs), antisense to targets, which bind to nematode-specific argonautes (WAGO) to bring about silencing (Bagijn et al., 2012). Second, piRNAs are produced from monocistronic genomic loci, the vast majority of which are preceded by a GTTTC consensus motif (the Ruby motif) (Ruby et al., 2006) that recruits RNA polymerase II (Pol II) to produce a 5′ capped 28-nt precursor transcript (Billi et al., 2013, Gu et al., 2012). Two piRNA clusters on chromosome IV, spanning 2.5 Mb and 3.7 Mb, contain over 90% of piRNA loci (Ruby et al., 2006). Pol II transcription of piRNA precursors requires the nematode-specific pseudokinase PRDE-1 (Weick et al., 2014) and the small nuclear RNA (snRNA) activating protein complex (SNAPc) component SNPC-4 (Kasper et al., 2014). SNPC-4 and PRDE-1 form a complex and together have been hypothesized to establish a specific chromatin structure essential for piRNA biogenesis (Kasper et al., 2014).

Several key aspects of nematode piRNA production remain poorly understood. Among the outstanding problems are the evolutionary origin of the Ruby motif, how Pol II is controlled such that it transcribes only very short piRNA precursors, and how the clustering of piRNA loci into genomic regions contributes to piRNA production. Here, through a comparative analysis of piRNA promoter structure and genomic organization across nematodes, we obtain new insights into these questions. First, we uncover the evolutionary origin of the Ruby motif, showing that nematode piRNA transcription evolved from snRNA transcription. Second, we reveal two distinct modes of piRNA locus organization in nematodes, where piRNA loci either are found in high-density clusters within repressed H3K27me3 chromatin domains or are dispersed throughout the genome within actively transcribed genes. Third, we discover a downstream sequence signal at nematode piRNA loci, which is likely to promote Pol II pausing at piRNA loci to generate short piRNA precursors. By using CRISPR-mediated genome editing, we confirm that both the surrounding chromatin environment and Pol II pausing sequence signals determine the activity of piRNA loci in nematodes.

## Results

### The Ruby Motif Is an Ancient piRNA Regulatory Module

**Figure 1:**
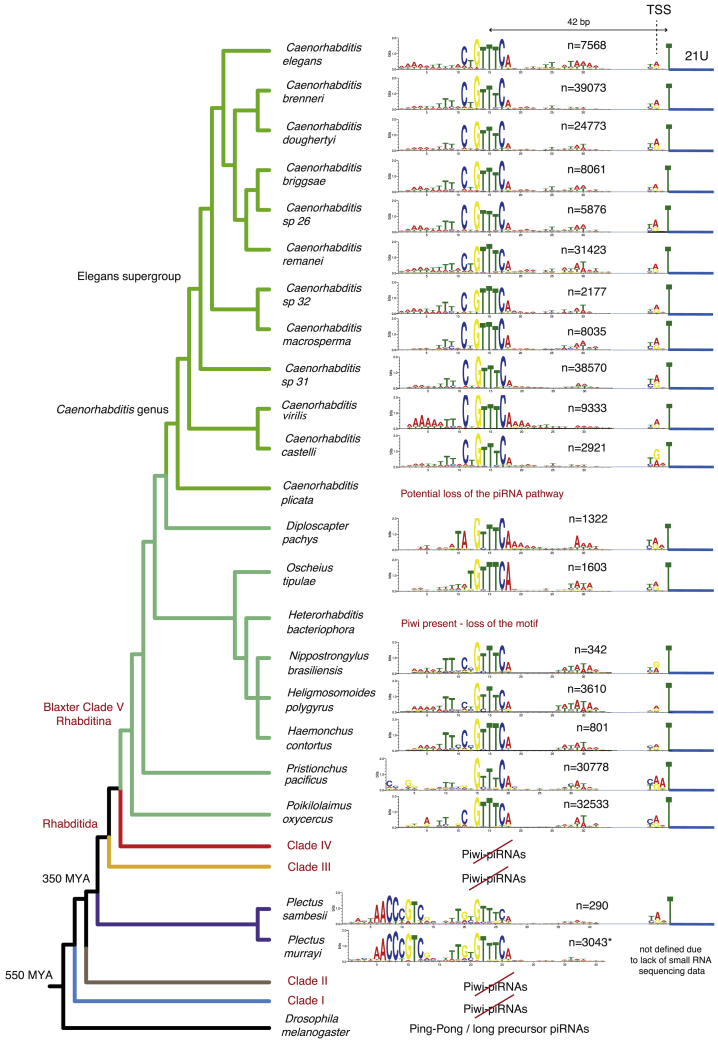
Overview of piRNA Evolution in Nematodes Assessment of Piwi protein presence/absence, Ruby motif presence/absence, and number of annotated piRNA loci. piRNA loci were defined as 21U-RNA read-mapping sites containing conserved upstream motifs, unless otherwise indicated. Nematode phylogeny was taken from Blaxter and Koutsovoulos, 2015. ^∗^*Plectus murrayi* piRNA loci were defined with genome-wide motif scans using the *P. sambesii* motif in the absence of small RNA sequencing (see STAR Methods).

We surveyed a wide taxonomic span of nematodes for components of the piRNA system. All of the nematodes we examined within Rhabditina (Clade V) possessed the Piwi protein PRG-1 (Figures 1 and S1A), with the exception of *Caenorhabditis plicata*, suggesting a recent loss of the piRNA pathway in this species. Outside of Clade V, we only identified Piwi in the closely related free-living nematodes *Plectus sambesii* and *Plectus murrayi* (plectids). The presence of Piwi in plectids, which are basal to Rhabditida (which includes Clades III, IV, and V), is consistent with the hypothesis that the piRNA pathway was lost independently in Clade III and Clade IV (Sarkies et al., 2015) (Figure 1).

To reveal the evolutionary dynamics of piRNA biogenesis, we used existing small RNA sequencing datasets (Sarkies et al., 2015, Shi et al., 2013) and sequenced a number of additional species (Figure 1). We sequenced and assembled *de novo* the genomes of *P. sambesii* and *Poikilolaimus oxycercus* and sourced other genomes from ongoing and published projects. In the majority of nematodes with Piwi, including in *P. sambesii*, we found a motif upstream of 21U-RNAs that bore remarkable similarity to the *C. elegans* core Ruby motif (Figure 1). We did not find any motifs upstream of any other small RNA class of defined 5′ nucleotide and length. This suggests that the Ruby motif originated at least 350 million years ago (mya) (Figure 1). Surprisingly, there was no evidence of upstream motifs in *Heterorhabditis bacteriophora* despite the presence of Piwi, suggesting that that the Ruby motif has been lost in this species. In all nematodes with Ruby-motif-dependent piRNAs, transcription initiated two nucleotides upstream of the mature piRNA (Figure S1B), just as in *C. elegans* (Billi et al., 2013, Gu et al., 2012, Weick et al., 2014). We observed large variability in the number of piRNA loci across genomes (Figure 1) that could not be attributed to differences in sequencing depth. The biological and evolutionary correlates of these differences await future study.

### The Ruby Motif Is Evolutionarily Related to the Nematode SNAPc-Binding Motif

**Figure 2:**
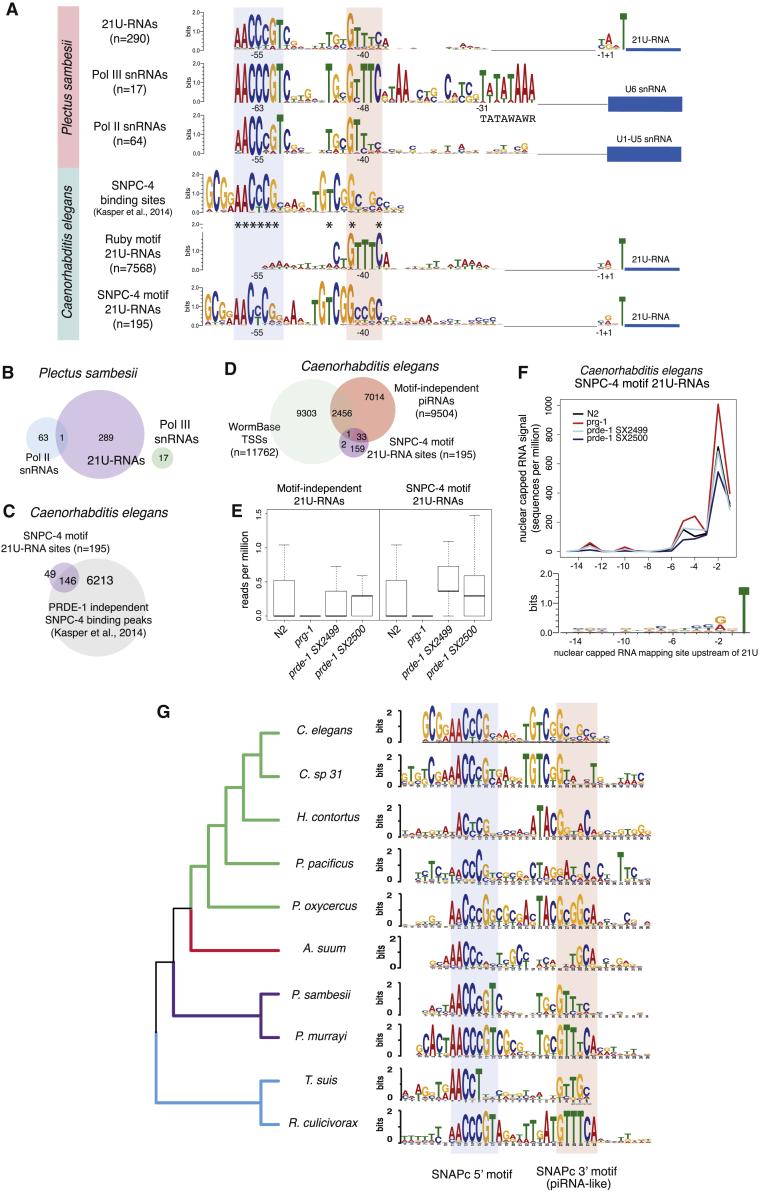
The Nematode piRNA Upstream Motif Evolved from the SNAPc-Binding Motifs (A) Sequence motifs upstream of piRNA and snRNA loci in *C. elegans* and *P. sambesii*. Stars indicate sequence matches between the *C. elegans* SNAPc binding motif and the *P. sambesii* piRNA motif. (B) Minimal overlap between *P. sambesii* piRNA and snRNA loci. (C) Overlap between SNPC-4 motif 21U-RNA loci and SNPC-4 binding sites detected by ChIP-seq analysis (Kasper et al., 2014). (D) Overlap between SNPC-4 motif 21U-RNA loci, motif-independent piRNAs (Batista et al., 2008), and WormBase transcription start sites of protein-coding genes (Chen et al., 2013). (E) Expression of motif-independent piRNAs and SNPC-4 motif 21U-RNA loci in N2 controls, and *prg-1* and *prde-1* mutant backgrounds. Boxplots show a line at the mean, the box represents the interquartile range and the whiskers extend to the furthest datapoint no more than 1.5 times away from the interquartile range. (F) Distribution of short nuclear-capped RNA mapping sites relative to 21U sequences at SNPC-4 motif 21U-RNA loci. Sequence composition analysis demonstrates the presence of YRNT initiator motifs. (G) Evolution of SNPC-4 motifs defined upstream of annotated snRNA loci across nematodes with and without piRNAs.

In *P. sambesii* and *P. murrayi*, the Ruby motif was accompanied by an additional 5′ motif (Figure 1). The two motifs almost always occurred together (Figure S1C). This extra motif bears a striking similarity to the *C. elegans* SNAPc motifs, predicted from binding sites of the SNAPc subunit SNPC-4 (Kasper et al., 2014) (Figure 2A). In *C. elegans*, SNPC-4 binds across piRNA cluster regions, but no enrichment relative to piRNA TSSs was detected (Kasper et al., 2014).

We defined SNAPc-binding motifs upstream of snRNA genes in *P. sambesii*. snRNA and piRNA promoters are drawn from distinct sets of loci but are highly similar both in their sequence motifs and their positioning relative to TSSs (Figures 2A, 2B, S2A, and S2B). While Pol III-dependent snRNA loci possessed a TATA-box, this was not found at either Pol II snRNA or piRNA loci, consistent with Pol II transcription of piRNAs (Hung and Stumph, 2011) (Figure 2A). This analysis suggests that direct SNPC-4 binding to piRNA promoters drives Pol II piRNA transcription in *P. sambesii* and *P. murrayi*.

We wondered whether SNPC-4 binding is sufficient for piRNA expression in *C. elegans*. We identified 297 21U read-mapping sites that contained SNPC-4 motifs defined by ChIP-seq at a distance that matched the positioning of the *P. sambesii* piRNA motif relative to TSSs (Figure 2A). The SNPC-4 binding motifs found upstream of 21U-RNAs show strong similarity to the motifs found upstream of snRNA loci in *C. elegans* (Figure 2C). After excluding sites overlapping with snRNA or snoRNA loci, 146 out of 195 sites overlapped with SNPC-4 binding sites identified by ChIP-seq in mutants lacking the dedicated piRNA biogenesis factor *prde-1* (Kasper et al., 2014) (Figure 2C). This suggests that SNPC-4 can be recruited to these sites independent of *prde-1* binding. The 21U-RNAs expressed from these sites significantly overlapped with previously annotated “Type II” piRNAs that are expressed independently of the Ruby motif (Figure 2D) and are present in *prde-1* mutants but undetectable in *prg-1* mutants (Figure 2E). Furthermore, these loci contained conserved YRNT motifs, and short-capped RNAs mapping 2 nt upstream of the 21U sequences (Figure 2F). Thus, we conclude that SNPC-4 binding independently of PRDE-1 can drive transcription of piRNAs in *C. elegans*, highlighting the functional similarities between snRNA and piRNA promoters.

We predicted SNAPc motifs in a wider sampling of nematode genomes, many of which have lost the piRNA pathway (Sarkies et al., 2015). Alignment of these motifs showed strong conservation of the 5′ half of the motif, while the 3′ half was more divergent (Figure 2G; Data S2 for an extended tree). The Clade I nematode *Romanomermis culicivorax*, which has lost the piRNA pathway altogether, possessed a conserved GTTTC site in the 3′ half of the SNAPc motif, resembling strongly the *P. sambesii* and *P. murrayi* piRNA motifs (Figure 2G). Ancestral sequence reconstruction suggested that the ancestral nematode SNAPc motif contained a GTTTC site in its 3′ half (Figure S3).

We conclude that snRNA and piRNA promoters are evolutionarily related. Given the ancestral nature of the GTTTC motif in snRNA promoters, the most likely evolutionary scenario is that the Ruby motif was co-opted from the 3′ half of an ancestral SNAPc motif, and the piRNA motif and SNAPc motif diverged subsequently in *Rhabditida* (Clades III, IV, and V).

### Two Levels of piRNA Clustering in Nematodes

**Figure 3:**
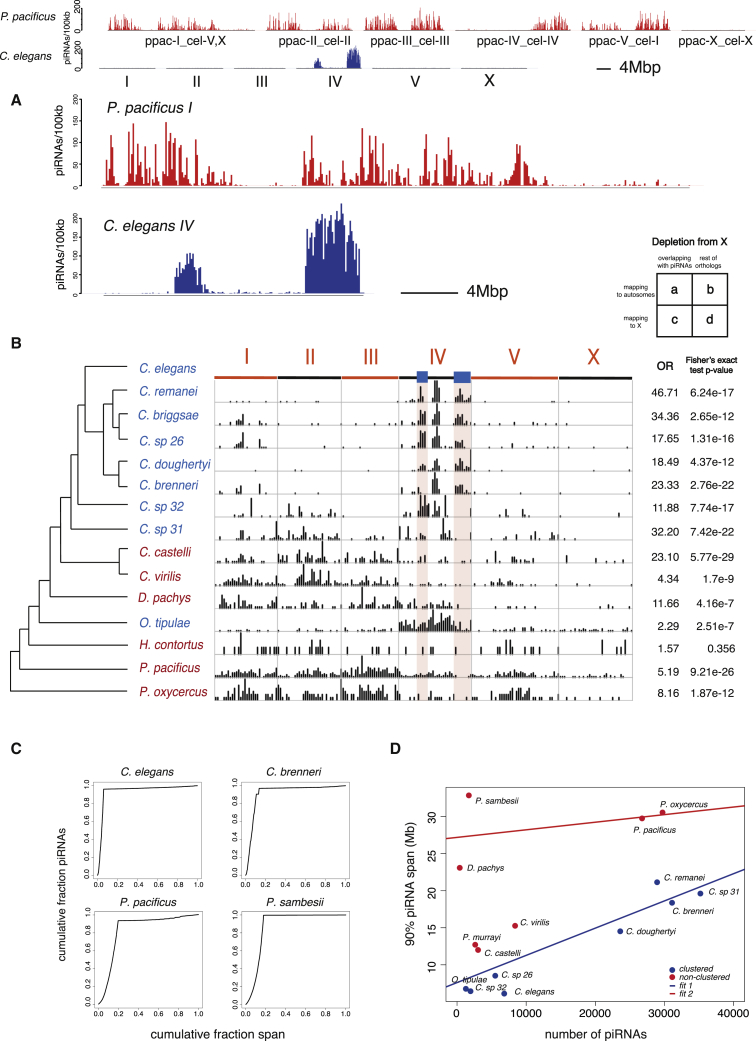
Two Modes of piRNA Locus Organization in Nematodes (A) Genomic distribution of piRNAs in *P. pacificus* (dispersed piRNAs) and *C. elegans* (clustered piRNAs). (B) Mapping of piRNA regions in multiple nematode species to the *C. elegans* genome. Odds ratio (OR) and Fisher’s exact test p value for X chromosome depletion is tabulated on the right-hand side. Species in red show broad mapping to *C. elegans* autosomes while species in blue show concentrated mapping to chromosome IV. (C) Cumulative number of piRNAs against cumulative span of contigs ranked by the significance of piRNA enrichment. Examples of clustered species (*C. elegans*, *C. brenneri*) and non-clustered species (*P. pacificus*, *P. sambesii*). (D) Discrimination plot of nematode genomes based on the number of piRNAs and the span of piRNA regions. Species were classified as clustered (blue dots) or non-clustered (red dots) on the basis of mapping of piRNA regions to *C. elegans* in (B). Best-fit lines are from bimodal regression analysis using all points as input.

piRNA loci controlled by Ruby motifs in *C. elegans* are almost exclusively located in two ∼3-Mb regions on chromosome IV. However, in the chromosomal-level genome assembly of *Pristionchus pacificus* (Rödelsperger et al., 2017), piRNA loci are distributed relatively evenly across the five autosomes but absent from the X chromosome (Figure 3A). *Pristionchus* is also in Rhabditina (Clade V) but is a member of Diplogasteromorpha, while *C. elegans* is in the Rhabditomorpha.

Most other nematode genome assemblies are of lower quality than those of *C. elegans* and *P. pacificus*. To analyze species with more fragmented genome assemblies, we developed and validated a method to identify piRNA locus-dense contigs (Figures S4A and S4B). We then used 1:1 orthologous protein coding genes to map the contigs to the *C. elegans* genome. We observed two large blocks of piRNA regions that contained orthologs that mapped to *C. elegans* chromosome IV in the majority of *Caenorhabditis* species and also in *O. tipulae* (blue in Figure 3B). This pattern suggested that the *C. elegans* piRNA clusters were conserved in these species. In other nematodes, including *P. pacificus*, regions with elevated density of piRNA loci mapped to several *C. elegans* chromosomes (red in Figure 3B), indicating that piRNA clusters are not conserved. Interestingly, genes mapping to the *C. elegans* X chromosome were depleted of piRNAs across all nematodes (Figure 3B). We confirmed a depletion of piRNAs on the X chromosome using independent X chromosome mappings in *O. tipulae*, where sex-linked genes have been used to identify the X chromosome (Besnard et al., 2017), *H. contortus*, for which there is a recent chromosomal assembly, and in the chromosomal assembly of *P. pacificus* (Figure S4C).

We quantified piRNA clustering by examining the extent to which piRNA loci were concentrated within genomic regions (Figure 3C). Bimodal regression analysis identified two groups, one with more clustered piRNAs and one with less clustered piRNAs. These groups were congruent with those recovered from mapping to orthologous regions in *C. elegans* (Figure 3D). On the basis of these analyses, we propose that there are two distinct modes of organization of piRNA loci in nematodes, one clustered, similar to *C. elegans*, and one more dispersed, similar to *P. pacificus*.

### Clustered and Dispersed piRNA Loci Have Distinct Chromatin Environments

To further characterize the differences between clustered and non-clustered modes of piRNA gene organization, we examined the chromatin environment of piRNA loci in *C. elegans* and *P. pacificus*. The *C. elegans* genome is organized into mutually exclusive, stable domains of H3K27me3 repressive chromatin (“regulated domains”) and H3K36me3 transcriptionally active chromatin (“active domains”) (Evans et al., 2016). While these domains were defined based on analyses of early embryo and L3 larval chromatin modifications (Evans et al., 2016), they are thought to be established in the germline (Rechtsteiner et al., 2010), and we found that H3K27me3 and H3K36me3 patterns are similar in adults (Figure S5A), when piRNAs are expressed (Das et al., 2008).

**Figure 4:**
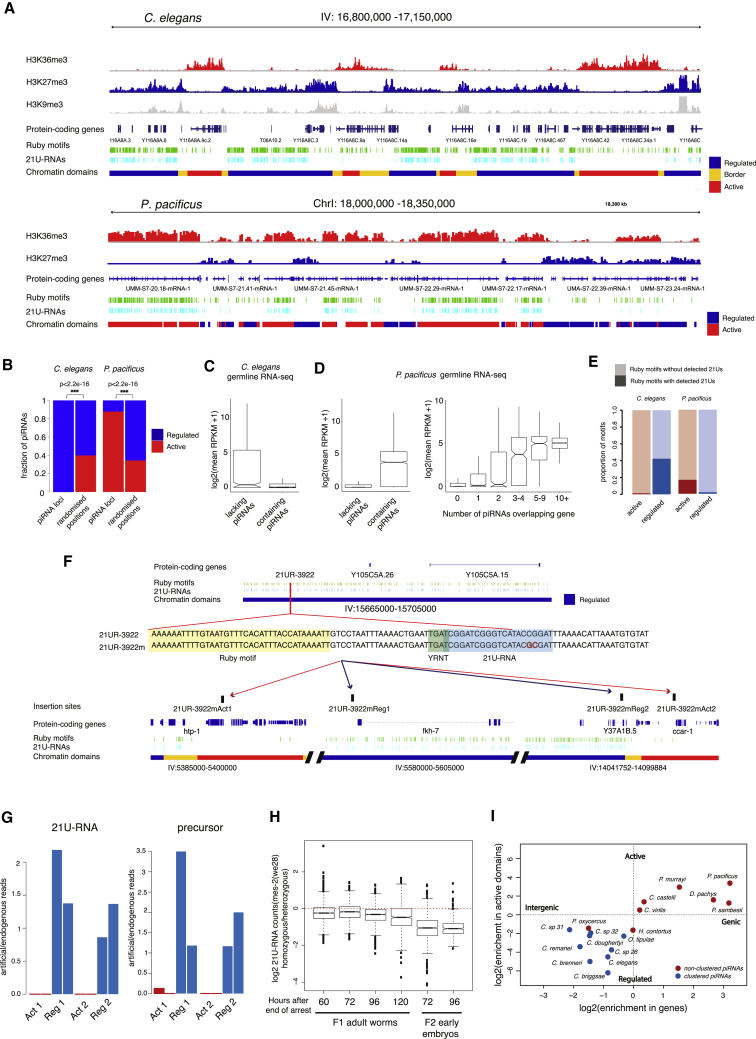
piRNAs in *C. elegans* and *P. pacificus* Occupy Different Chromatin Environments (A) *C. elegans* piRNA cluster regions are organized in multiple subclusters of piRNAs associated with H3K27me3 chromatin and interrupted by H3K36me3 chromatin domains depleted of piRNAs. *P. pacificus* piRNA loci are found within the introns of genes within H3K36me3 chromatin domains. (B) Quantification of chromatin domain locations of piRNAs in *C. elegans* and *P. pacificus* relative to the proportions expected from a uniform distribution of piRNA loci across the genome. (C) Germline expression of *C. elegans* protein-coding genes overlapping with piRNAs and lacking piRNAs. Boxplots show a line at the mean, the box represents the interquartile range and the whiskers extend to the furthest datapoint no more than 1.5 times away from the interquartile range. (D) Germline expression of *P. pacificus* protein-coding genes overlapping with piRNAs and lacking piRNAs and stratified according to their piRNA content. Boxplots show a line at the mean, the box represents the interquartile range and the whiskers extend to the furthest datapoint no more than 1.5 times away from the interquartile range. (E) Fraction of Ruby motifs associated with detectable 21U-RNAs in different chromatin environments in *C. elegans* and *P. pacificus*. (F) Design and generation of artificial piRNA loci by genome-editing. (G) Expression of an artificial piRNA placed in either regulated or active chromatin. 21U-RNA and precursor reads detected from the artificial locus were normalized to those of the endogenous locus. The two bars for each genomic site represent independent strains. (H) Log_2_ fold-change in piRNA abundance in mes-2(we28) homozygous *C. elegans* worms relative to heterozygous siblings at several time points during adulthood after end of L1 starvation. Early embryo samples measure maternal contribution of piRNAs in both genotypes. Boxplots show a line at the mean, the box represents the interquartile range and the whiskers extend to the furthest datapoint no more than 1.5 times away from the interquartile range. (I) Two fundamental modes of piRNA organization in nematodes. Discrimination plot based on genic/intergenic proportions of piRNAs and chromatin association of piRNAs. Chromatin domains were defined based on mappings of 1:1 orthologs to *C. elegans*. Species are colored according to the designation in Figure 3.

Within the two piRNA clusters on chromosome IV, 95% of piRNA loci overlapped with H3K27me3/regulated domains (Figures 4A and 4B). Indeed, out of 9,634 21U-RNA sequences within cluster regions, only 4 were produced from H3K36me3/active domains. This represents a 286-fold depletion of piRNA loci from active chromatin domains compared to a uniform distribution across piRNA cluster regions (Fisher’s exact test p < 2.2e−16). In addition, regulated domains were significantly larger within piRNA cluster regions compared to the rest of the genome (Figure S5B). Genes containing piRNA loci showed extremely low or null expression in the germline, while genes located in cluster regions but lacking piRNA loci were highly expressed (Wilcox unpaired test p < 2.2e−16, Figure 4C). Together, these data strongly suggest that expression of *C. elegans* piRNAs occurs predominantly from regulated domains enriched in H3K27me3.

The organization of piRNA loci in *P. pacificus* contrasts with that of *C. elegans* (Figures 4A and 4B). 88% of *P. pacificus* piRNA loci were localized within protein-coding genes (1.6-fold enrichment within genes relative to that expected by chance, p < 0.01 simulation test, Figure S5C), while only 45% of *C. elegans* piRNAs were found within genes (1.34-fold depletion from genes, p < 0.01 simulation test; Figure S5C). *P. pacificus* piRNA loci are distributed approximately equally in the sense and antisense orientation of the genes in which they are found. 97% of the intragenic piRNA loci are found within introns (empirical p < 0.01 simulation test, Figure 4A). The chromatin environment of *P. pacificus* piRNAs also contrasts with that of *C. elegans*. We defined active and repressive chromatin domains in *P. pacificus* using publicly available ChIP-seq data (Werner et al., 2018). 88% of piRNA loci overlapped with H3K36me3 active domains (2.5-fold enrichment, FET p < 2.2e−16), while only 12% localized to domains of H3K27me3 (5.4-fold depletion, FET p < 2.2e−16) (Figures 4A and 4B). To further confirm this result, we profiled gene expression from *P. pacificus*-dissected gonads. Consistently, protein-coding genes overlapping with piRNAs have high germline expression (Figure 4D). Stratification of protein-coding genes according to their piRNA content showed a trend of increasing expression with increased piRNA content (Figure 4D).

We examined the fraction of Ruby motifs associated with expressed 21U-RNAs in *C. elegans* and *P. pacificus* in different chromatin environments. In *C. elegans*, 74% of Ruby motifs within regulated chromatin were associated with detectable 21U-RNAs, contrasting with less than 2% of those in active chromatin (Figures 4E and S5D). In *P. pacificus*, 20% of Ruby motifs within predicted active domains were associated with 21U-RNAs, compared to 2% of motifs in regulated domains (Figures 4E and S5D). The fraction of piRNA loci with motifs expressed in *P. pacificus* was positively correlated with the expression of the overlapping protein-coding genes (Figure S5E), suggesting that host protein-coding gene expression promotes expression of the 21U-RNAs.

We used CRISPR-Cas9 genome editing in *C. elegans* to test the role of chromatin in piRNA biogenesis. We selected an endogenous piRNA locus (21UR-3922) and modified the 21U sequence to generate an artificial piRNA that could be distinguished from all endogenous piRNAs by sequencing (21UR-3,922m; Figure 4F). We inserted the artificial piRNA gene into regulated and active chromatin domains in each of the two clusters in chromosome IV (Figure 4F). The insertion had no effect on global piRNA levels (Figures S6A and S6B). When inserted into regulated chromatin regions, the artificial piRNA was expressed at similar levels to the endogenous piRNA (Figure 4G). However, there was no detectable expression of the mature 21U-RNA when inserted into either of the active chromatin regions (Figure 4G).

To further test the role of chromatin in piRNA biogenesis, we sequenced piRNAs from *C. elegans mes-2* mutants, which lack H3K27me3 in the germline. Although H3K27me3 is essential for germline formation, maternally provided *mes-2* protein supports germline development for a single generation after the onset of homozygosity, allowing us to assay piRNA production in the absence of H3K27me3 (Figure S6C). We found a decrease in 21U-RNA and piRNA precursor levels in homozygous *mes-2(we28)* worms, in which the locus is deleted, compared to their heterozygous siblings (Figures 4H, S6D and S6E). In addition, we observed a specific decrease in motif-dependent 21U-RNA and precursor abundance relative to motif-independent piRNAs expressed from active chromatin (Figures S6F and S6G). Altogether, our data suggests that piRNAs must be located within regulated chromatin domains enriched with H3K27me3 in order to be transcribed in *C. elegans*.

To generalize these observations, we assessed the intergenic and genic proportions and the predicted chromatin environment of piRNA loci across nematodes. We predicted the chromatin locations of protein-coding genes across nematodes based on the chromatin locations of their *C. elegans* 1:1 orthologs. *P. pacificus* chromatin domain predictions were highly consistent with ChIP-seq defined domains, and with germline expression, validating the approach (Figure S7; STAR Methods). Species could be divided into two groups, one with more genic piRNAs associated with predicted active chromatin domains, and one with more intergenic piRNAs associated with predicted regulated chromatin domains. These categories overlapped strongly with the pattern of clustering. Species with clustered piRNAs had H3K27me3/regulated-enriched piRNAs, while less clustered species had H3K36me3/active enriched piRNAs (Figure 4I).

We propose that there are two fundamental modes of piRNA organization in nematodes. “Caenorhabditis”-type (C-type) piRNAs are organized into dense clusters within repressive chromatin. These sub-clusters are then grouped together into a large “super-cluster,” for example, the regions on chromosome IV in *C. elegans*. In contrast, “Pristionchus”-type (P-type) piRNAs are more widely distributed across the genome where they are found within the introns of germline-expressed genes, enriched for H3K36me3.

### Chromatin Domain Organization of piRNAs Is under Selection in Nematodes

**Figure 5:**
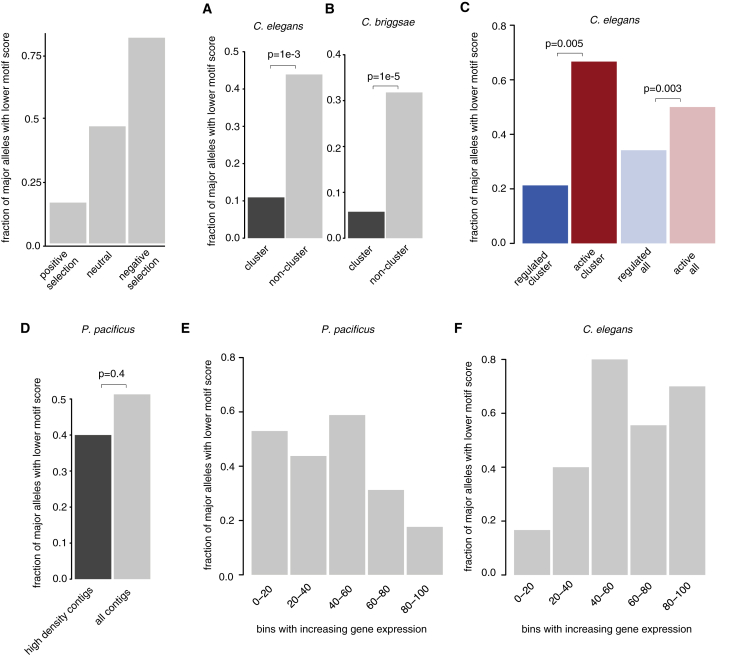
Selection at piRNAs Is Dependent on Their Chromatin Environment (A and B) Fraction of major alleles with lower Ruby motif score (present in >90% of strains) in *C. elegans* (A) and *C. briggsae* (B). The p values for these and the rest of subfigures are derived from a Fisher’s exact test to compare the proportions. (C) Fraction of major alleles with lower Ruby motif score across different chromatin environments in *C. elegans*. (D) Fraction of major alleles with lower Ruby motif score in regions of low and high piRNA density in *P. pacificus*. (E and F) Fraction of major alleles with lower Ruby motif score in genes stratified according to the expression in *C. elegans* and *P. pacificus*.

The different modes of piRNA organization led us to ask how selection is acting on them. Using wild-isolate, genome-wide single nucleotide polymorphism (SNP) data from the *C. elegans* Natural Diversity Resource (Cook et al., 2017), *C. briggsae* (Thomas et al., 2015), and *P. pacificus* (Rödelsperger et al., 2014), we explored the predicted effects of SNPs on Ruby motifs by measuring their effect on the match of the motifs to a consensus position weight matrix. The allele frequency spectrum was markedly different between motifs within the piRNA locus clusters and those outside for both *C. elegans* and *C. briggsae* (Figures S8A and S8B). In *C. briggsae* and *C. elegans*, alleles with a low motif score were much less likely to be the major allele (present in >90% of strains) within the cluster than outside the cluster, implying that piRNAs are under stronger selection within the cluster than outside (p < 1e−3 for both, Fisher’s exact test, Figures 5A and 5B). Similarly, both within and outside the cluster, selection was stronger on piRNAs within H3K27me3 domains than H3K36me3 domains (Figure 5C).

In *P. pacificus*, observed SNPs tended to have a smaller effect on the motif score (Figure S8C), perhaps reflecting the lower information content within the motif (Figure 1). The major allele frequencies of low-scoring motifs did not vary with the density of piRNA loci, consistent with the lack of clusters (Figure 5D). However, in *P. pacificus*, SNPs predicted to disrupt piRNAs were less likely to be the major allele in highly expressed genes compared to lowly expressed genes (Figure 5E). Exactly the opposite trend was observed in *C. elegans* (Figure 5F). Together, these data confirm that the two different modes of piRNA biogenesis are under selection in their respective species.

### Comparative Analysis Implicates RNA Pol II Pausing in piRNA Production in Nematodes

A puzzling feature of piRNA biogenesis in nematodes is how Pol II is regulated to produce the ∼30 nt precursor of mature piRNAs (Gu et al., 2012). Our finding that piRNAs can be found in both repressed and active chromatin environments is challenging, as *Pristionchus*-like piRNAs localize to regions of high transcriptional activity expected to favor elongating Pol II. Pol II is known to pause 20–65 nucleotides downstream of the transcription start site (TSS) at certain types of eukaryotic protein-coding gene promoters. This results in the production of short-capped RNAs that are rapidly degraded by the nuclear exosome. This has been proposed as the underlying mechanism of piRNA transcription in nematodes (Gu et al., 2012), but so far evidence has been lacking.

Previous genome-wide analyses in *D. melanogaster* identified a sequence signature associated with promoter-proximal Pol II pausing, characterized by a region with high melting temperature (T_m_) relative to the genome-wide background and a region with low T_m_ immediately downstream (Nechaev et al., 2010). Low T_m_ of the RNA-DNA hybrid at the active site of Pol II is known to destabilize the elongation complex (Kireeva et al., 2000), causing Pol II to pause (Gressel et al., 2017). This is thought to lead to backtracking of Pol II from the low T_m_ region to the high T_m_ region further upstream where RNA-DNA interactions are more stable. Consequently, Pol II pausing is observed at the boundary between the high T_m_ and low T_m_ regions.

**Figure 6:**
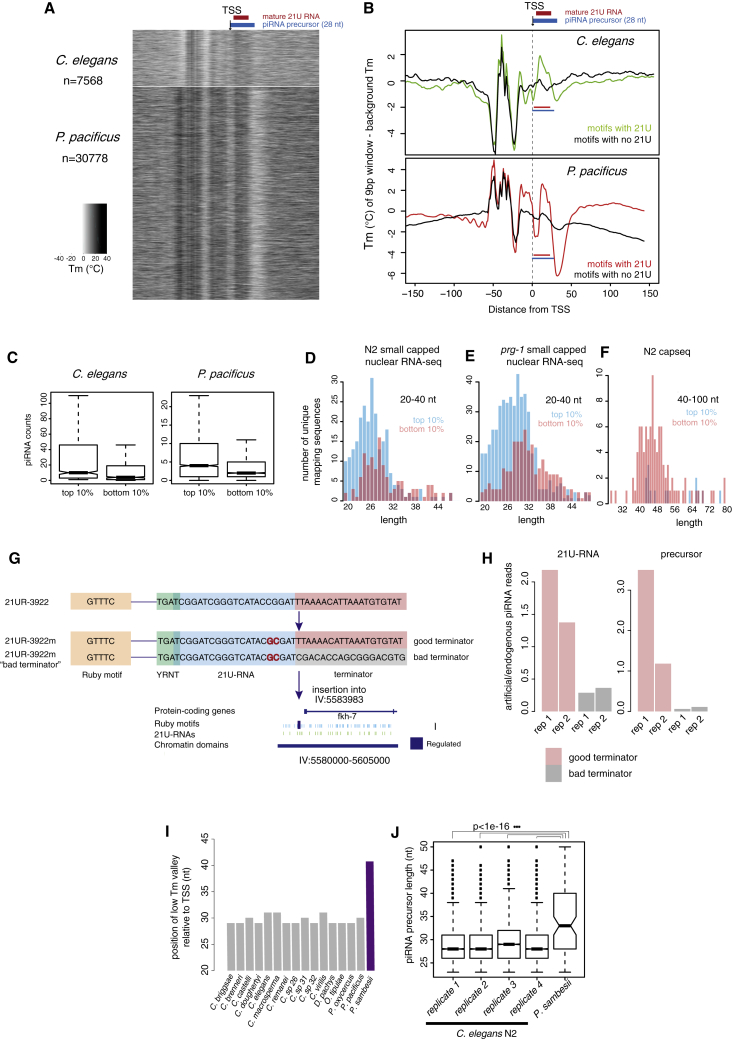
Transcription of Short-Capped piRNA Precursors in Nematodes Occurs through Pausing of Pol II (A and B) Melting temperature profiles around piRNA transcription start sites in *C. elegans* and *P. pacificus*. (C) Relationship between the strength of pausing-associated sequence signatures and mature piRNA levels. Boxplots show a line at the mean, the box represents the interquartile range and the whiskers extend to the furthest datapoint no more than 1.5 times away from the interquartile range. (D and E) Length and abundance of piRNA precursors produced from loci with strong (top 10%) or weak (bottom 10%) pausing-associated signatures in *C. elegans* assessed by high-throughput sequencing of capped RNAs less than 50 bp in size. (D) shows WT and (E) shows *prg-1* mutants. (F) Longer nuclear-capped RNAs (40–60 bp) emanating from piRNA loci with strong (top 10%) or weak (bottom 10%) pausing-associated sequence signatures assessed by capseq (Gu et al., 2012); compare with (D) and (E). (G) Design and generation of artificial piRNA loci with good and bad terminator sequences. (H) Expression of the artificial piRNA locus with good and bad terminators. 21U-RNA and precursor reads detected from the artificial locus were normalized to those of the endogenous locus. (I) Location of the low melting temperature region downstream of piRNA loci. The distance to the center of the low T_m_ “valley” from the piRNA transcription start site is shown. (J) piRNA precursor length in *C. elegans* and *P. sambesii*. Boxplots show a line at the mean, the box represents the interquartile range and the whiskers extend to the furthest datapoint no more than 1.5 times away from the interquartile range.

We examined T_m_ signatures around nematode piRNA TSSs. *P. pacificus* piRNA loci displayed a strong match to the pausing-associated sequence signature, such that a marked reduction in T_m_ relative to the genome-wide background coincided exactly with the predicted 3′ ends of piRNA precursors (Figures 6A, 6B, S9A and S9B). Sequence motifs have not previously been identified downstream of *C. elegans* piRNAs (Ruby et al., 2006); nevertheless we found a clear match to the pausing-associated signature at *C. elegans* piRNA loci, although this was less pronounced than in *P. pacificus* (Figures 6A, 6B, S9A, and S9B).

We then examined the role of the pausing-associated sequence signature on piRNA expression by stratifying piRNA loci according to the strength of this signature (see STAR Methods). In both *C. elegans* and *P. pacificus*, mature 21U-RNA expression was significantly greater from the loci with the strongest pausing-associated signatures than from the weakest (p < 1e−10 Wilcoxon unpaired test) (Figure 6C). The GC content of the 21U-RNA is not responsible for this effect, as sequence downstream of the 21U itself alone had a significant impact on mature piRNA abundance (Figure S9C).

To test the role of the pausing signal on piRNA transcription, we examined *C. elegans* piRNA precursors using nuclear short-capped RNA sequencing data from a previous study (Weick et al., 2014). piRNA precursors produced from loci with strong pausing signals were significantly shorter and on average 2-fold more abundant than those produced from loci with weak pausing signals (Figures 6D and S9D–S9H). This effect was emphasized in *prg-1* mutants, where mature piRNAs are not present, leading to accumulation of piRNA precursors in sequencing data (Figures 6E and S9D–S9H). Consistently, the few atypical longer precursors (40–100 bp) sequenced by capseq (Gu et al., 2012) mapped almost exclusively to loci with the weakest pausing signals (Figures 6F and S9D–S9H).

We modified the sequence downstream of the artificial 21UR-3,922m to increase the T_m_ by 27.5°C, and inserted this new artificial piRNA (“21UR-3,922 m-bad terminator”) into one of the regulated domain sites (IV:5583983) (Figure 6G). This allowed us to compare the expression of two piRNA loci that have different pausing signal strength but are otherwise identical. The 21UR-3922m with the higher downstream T_m_ was expressed at around 4.5-fold lower levels than the original artificial 21UR-3,922 m with the same sequence on the same genomic site (Figure 6H). Moreover, the piRNA precursor levels were ∼15-fold lower (Figure 6H). Other piRNAs were not affected (Figure S9I). Thus, the T_m_ of the downstream sequence alone is sufficient to affect transcription of the piRNA locus.

We detected similar Pol II pausing-associated sequence signatures across nematodes (Figures S10A–10C). In *P. sambesii*, the low T_m_ region downstream of the 21Us is shifted ∼10 nt further downstream compared to the rest of nematodes examined (Figures 6I and S10C). Compared with *C. elegans*, piRNA precursors are significantly longer in this species (Figure 6J), consistent with a role for the pausing signal in piRNA transcription termination.

**Figure 7:**
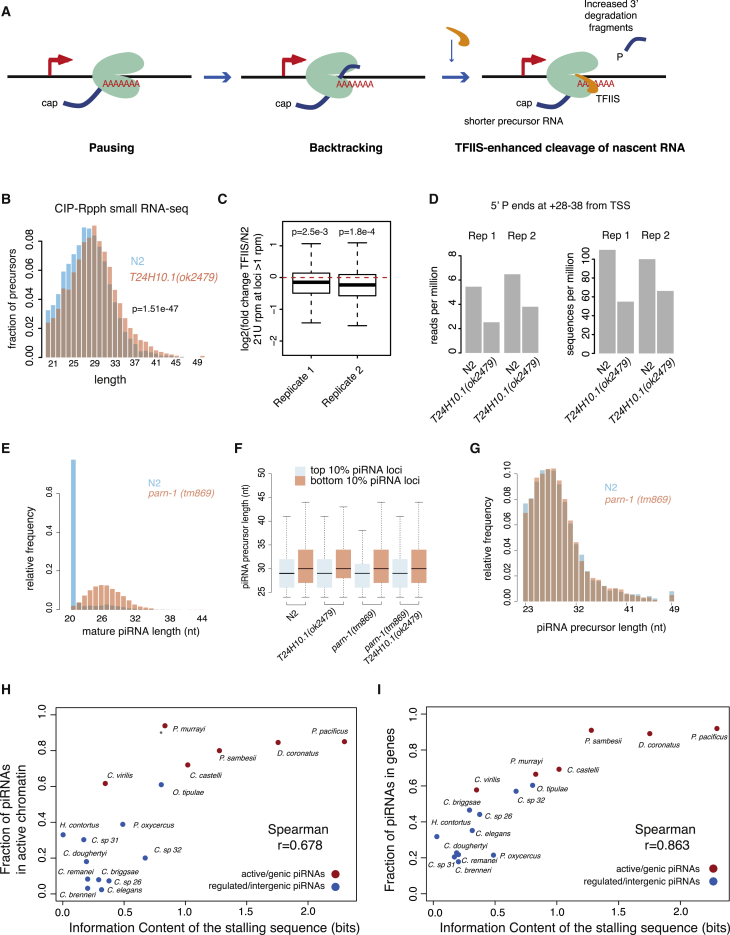
Chromatin and Pol II Pausing Together Control Nematode piRNA Biogenesis (A) The role of TFIIS in modulating promoter-proximal pausing. Upon pausing and backtracking, TFIIS stimulates cleavage of the 3′ end of the nascent RNA, leading to shorter short-capped RNAs and the release of degradation fragments. (B) piRNA precursor length in TFIIS mutants (*T24H10.1(ok2479)*) compared to wild-type. (C) Mature piRNAs in TFIIS mutants (*T24H10.1(ok2479)*) compared to wild-type. Boxplots show a line at the mean, the box represents the interquartile range and the whiskers extend to the furthest datapoint no more than 1.5 times away from the interquartile range. (D) 3′ degradation fragments of piRNA precursors in TFIIS compared to WT *C. elegans*. 3′ degradation fragments are defined as 5′ monophosphate small RNAs whose 5′ end maps between 28 and 38 nt downstream of the piRNA transcription start site. (E) Length distribution of 5′ processed piRNAs in N2 compared to *parn-1(tm869)* mutants. (F) Length distribution of 5′ unprocessed piRNA precursors, comparing the top and bottom 10% of piRNA loci stratified according to the strength of the pausing signal, in different mutant backgrounds. (G) Length distribution of 5′ unprocessed piRNA precursors in wild-type nematodes compared to *parn-1(tm869)* mutants. (H and I) Relationship between the average strength of pausing signal and enrichment of piRNAs in active chromatin (H) and enrichment of piRNAs in genic regions (I) across different nematode species. Boxplots show a line at the mean, the box represents the interquartile range and the whiskers extend to the furthest datapoint no more than 1.5 times away from the interquartile range.

To test the involvement of pausing in piRNA production, we examined *C. elegans* mutants carrying a deletion in TFIIS. TFIIS is a general transcription elongation factor that rescues backtracked Pol II complexes by promoting cleavage of the 3′ end of the nascent RNA by Pol II itself (Schweikhard et al., 2014). In *D. melanogaster*, TFIIS deletion leads to an increase in the length of promoter-associated short-capped RNAs, indicative of pausing and backtracking of Pol II at the promoter (Nechaev et al., 2010) (Figure 7A). *C. elegans* mutants carrying a deletion in the ortholog of TFIIS (*T24H10.1*(*ok2479*)) showed significantly longer piRNA precursors (Figures 7B and S11A–11C) and a modest, but consistent, decrease in mature piRNA abundance (Figure 7C). The effect of the TFIIS deletion is specific for short-capped RNAs, as we did not find differences in length distributions of rRNA degradation fragments (Figures S11D–11G) or tailed 22G-RNAs (Figure S11H). To test whether TFIIS promotes the cleavage of piRNA precursors, we examined sequencing data for potential 3′ cleavage fragments, with a 5′ monophosphate mapping 28–38 nucleotides downstream of the piRNA TSS. We detected potential cleavage products from around 10% of loci in wild-type nematodes, but these products were 2-fold reduced in TFIIS mutants (Figure 7D). These data support a direct role for Pol II pausing in termination of piRNA transcription and release of piRNA precursors.

In *C. elegans*, piRNA precursors are trimmed by the exonuclease PARN-1, an ortholog of Trimmer from *B. mori* (Tang et al., 2016) (Figure 7E). An alternative explanation for our findings would be if the sequence signature exerted its effect post-transcriptionally by affecting the trimming of capped piRNA precursors. However, this is unlikely to be the case as capped piRNA precursor length differences between loci with strong and weak pausing signals remain unaffected in the absence of trimming (Figure 7F). In addition, the overall length of capped piRNA precursors does not change in *parn-1* (tm869) mutants compared to wild-type nematodes (Figure 7G). These data are consistent with cytoplasmic trimming on an uncapped Piwi-bound piRNA precursor, thus nuclear short-capped RNAs represent early unprocessed piRNA precursor transcripts.

Altogether, our data strongly suggest that the sequence properties of piRNA loci promote Pol II pausing and are thus important for the generation of short piRNA precursors.

### Synergy between Chromatin Environment and Pausing-Associated Sequence Signals in Nematode piRNA Biogenesis

Though clearly important for piRNA biogenesis, the Pol II pausing signature is weaker in *C. elegans* than in *P. pacificus*. To test whether this extended to the remaining C-type and P-type species, we quantified the strength of Pol II pausing-associated sequence signatures at piRNA loci across nematodes (Figure S10). Across nematode species, the strength of the Pol II pausing-associated sequence signature correlated positively both with the proportion of piRNAs in active chromatin and the proportion of piRNAs within genes (Spearman rho > 0.67 for both, p = 1.9e−3 and p = 3.89e−6; Figures 7H and 7I). Thus, C-type species, in which piRNA loci are found within H3K27me3 chromatin domains, have relatively weak pausing signals, whereas stronger pausing signals are found in P-type species where piRNA loci are found within H3K36me3 domains.

## Discussion

Our analysis of piRNA organization and biogenesis across nematodes not only illuminates the evolutionary history of the piRNA system in nematodes but also provides unexpected insights into the fundamental mechanism of piRNA production in *C. elegans*.

### Co-option of snRNA Promoter Elements for piRNA Biogenesis

**Figure 8:**
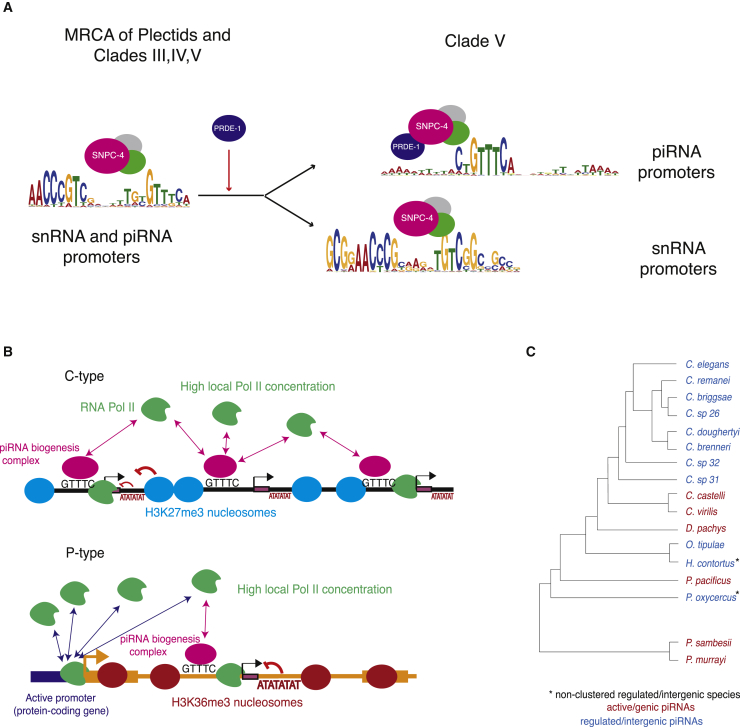
Evolution of piRNA Biogenesis in Nematodes (A) Model for the emergence of Ruby motif-dependent piRNAs by co-option of snRNA promoter elements and transcription factors. (B) Two modes of piRNA genomic organization in nematode genomes. (C) Widespread phylogenetic distribution of active-genic and regulated-intergenic piRNAs.

Our comparative genomic analyses demonstrate that the Ruby motif found upstream of nematode piRNAs is evolutionarily related to the motif found upstream of the U1-6 snRNA loci. This motif interacts with the SNAP complex via the DNA binding protein SNPC-4. In the nematodes *P. sambesii* and *P. murrayi*, the SNAPc and piRNA upstream motifs are almost identical. Indeed, our ancestral sequence reconstruction suggests that in the common ancestor of nematode Clades I–V, the SNAPc motif contained the characteristic GTTTC found in the Ruby motif. As SNAPc is conserved across eukaryotes, thus predates nematode piRNA biogenesis, the most likely evolutionary scenario is that the Ruby motif is derived from the motif controlling snRNA transcription (Figure 8A).

The similarity between the piRNA upstream motif and the SNAPc motif in *P. sambesii* and *P. murrayi* suggests that the snRNA machinery is directly involved in piRNA biogenesis in these nematodes. Across Clade V nematodes, only the 3′ half of the SNAPc motif, which contains the GTTTC site, is conserved at the vast majority of piRNA loci. Interestingly however, we find that a subset of piRNAs in *C. elegans* is downstream of the full SNAPc binding site. Moreover, even at piRNA loci with only the 3′ half of the motif, SNAPc is still required for piRNA biogenesis (Kasper et al., 2014). It is intriguing that loss of the 5′ half of the SNAPc motif upstream of piRNAs correlates with the appearance of the specialized piRNA biogenesis factor PRDE-1 (Figure S1A), which is known to interact with SNPC-4 (Kasper et al., 2014). We speculate that PRDE-1 may recruit SNPC-4 to piRNA loci in the absence of the 5′ half of the motif (Figure 8A). This may have evolved to enable separate regulation of piRNA and snRNA loci. It should be noted, however, that the nematode *O. tipulae* has apparently lost PRDE-1 (Figure S1A), so there may be additional mechanisms involved in this process.

Recent findings in Drosophila have shown that piRNA biogenesis involves a number of specific biogenesis factors derived from the canonical Pol II transcription machinery (Andersen et al., 2017). Similarly, we hypothesize that piRNAs in nematodes evolved through co-option of a pre-existing snRNA promoter element and its associated transcription factor. Recurrent repurposing of the transcription machinery for piRNA biogenesis may be an important theme facilitating rapid evolution in the arms race with TEs.

### RNA Pol II Pausing as a Key Feature of piRNA Production in Nematodes

Previous identification of piRNA precursors by deep sequencing in *C. elegans* demonstrated piRNA production by Pol II (Billi et al., 2013, Gu et al., 2012), and it was hypothesized that piRNA precursors might result from promoter-proximal Pol II pausing (Gu et al., 2012). This suggestion was prescient: our analysis of piRNA biogenesis across nematodes reveals that this process is indeed likely to be responsible for the short length of piRNA precursors.

Following transcription initiation, Pol II forms an early elongation complex with limited elongation capacity, requiring further regulation to transition into productive elongation. During early elongation, Pol II is highly susceptible to pausing, which can lead to backtracking by a few nucleotides (Schweikhard et al., 2014). At many promoters, Pol II rarely switches into elongation and is instead released from the template, resulting in the accumulation of short-capped RNAs derived from the promoter of the gene. We suggest that exactly this cycle of initiation, pausing, and premature termination results in the production of piRNA precursors. Our model is supported by recent studies demonstrating that initiation, pausing, and release of Pol II occurs continuously at paused promoters (Krebs et al., 2017, Steurer et al., 2018). The stability of promoter-proximal Pol II is dependent on the negative elongation factor NELF. Depletion of NELF leads to faster reinitiation-pausing cycles that result in the release of short-capped RNAs from chromatin (Henriques et al., 2013). *C. elegans* lacks a NELF ortholog, which may increase the rate of piRNA production through faster reinitiation-pausing cycles.

The involvement of Pol II pausing in production of piRNAs in *C. elegans* is consistent with other findings demonstrating production of functional small RNAs from promoter-proximal Pol II. Mammalian Ago2 binds to small RNAs produced from hairpins derived from paused Pol II at protein-coding gene promoters (Zamudio et al., 2014). In ciliates, Pol II pausing in conjunction with TFIIS activity generates ∼25 nt RNAs that are loaded into Piwi proteins (Gruchota et al., 2017). These observations suggest that Pol II pervasive initiation and pausing can be co-opted for the biogenesis of functional small RNAs across eukaryotic genomes, including mammals.

### Dynamic Evolution of the piRNA Pathway: Two Alternative Modes of piRNA Organization and Biogenesis across Nematodes

Here, we show that two modes of large-scale genomic organization of piRNAs are found in nematodes. In P-type species, piRNA loci are found within active chromatin and are not clustered together, as observed in *P. pacificus*. In contrast, C-type species have piRNAs clustered at high density in regions of H3K27me3-repressive chromatin. These are separated by regions of active H3K36me3 chromatin depleted of piRNAs; therefore, *C. elegans* piRNA clusters are better described as “clusters-of-clusters.” In all species, piRNAs are depleted from the X chromosome. This may reflect the fact that the X chromosome has a distinct chromatin structure from autosomes without clear H3K36me3 and H3K27me3 domains (Evans et al., 2016).

The two modes of organization may represent alternative strategies to achieve high local concentration of Pol II to support piRNA expression (Figure 8B). In P-type species, piRNA promoters localize within actively transcribed genomic regions, thus “piggybacking” on the capacity of protein-coding genes to attract the transcriptional machinery. In C-type species, the high density of piRNA loci may serve to attract sufficient Pol II to a region devoid of active genes.

While clearly required for piRNA expression, the mechanisms by which the H3K27me3-repressive domains promote piRNA transcription in *C. elegans* remain elusive. H3K27me3 might be required globally in piRNA regions to maintain silenced protein-coding genes and ensure selective transcription of piRNA genes. Alternatively, organization into H3K27me3 domains might play a role in the formation of a nuclear compartment where multiple H3K27me3 domains come close together in space. Whether H3K27me3 nucleosomes play a role at piRNA promoters locally remains unclear. It is possible that SNPC-4 and its associated cofactors induce chromatin remodeling at piRNA promoters upon recognition of the Ruby motif. Profiling of chromatin modification and accessibility in the germline will directly address these questions.

P-type and C-type piRNAs differ in their reliance on *cis*-acting Pol II pausing-associated sequence signatures downstream of the piRNAs: while these signatures are relatively weak at piRNA loci in C-type species, they are much more obvious at piRNA loci in P-type species. The H3K27me3 chromatin environment may play a direct role in promoting Pol II pausing in C-type species. In P-type species, a stronger pausing signal may be required to ensure correct termination due to increased chromatin accessibility within actively transcribed regions.

This model is consistent with genome-wide studies in a variety of model systems showing that H3K27me3 is anticorrelated with the elongating form of Pol II. It has been reported that H3K27me3 accumulation at promoters in the absence of H2BK119 mono-ubiquitination by PRC1, directly promotes short-capped RNA production at poised genes (Min et al., 2011). This is particularly interesting because *C. elegans* lacks a clear germline-expressed ortholog of PRC1 and thus likely does not have germline H2B-monoubiquitination in H3K27me3-rich regions (Karakuzu et al., 2009). However, this finding applies to bivalent genes enriched in H3K4me3 in addition to H3K27me3, and whether H3K27me3 alone is sufficient is unknown.

Currently, we have insufficient evidence to establish which mode of biogenesis was ancestral. It appears that the mode may switch dynamically. For example, *D. pachys* has P-type piRNAs despite being nested within the C-type species *C. elegans* and *O. tipulae* (Figure 8C). It is therefore possible that both types of piRNAs use common biogenesis machinery and could coexist within the same organism. In support of this, we note that some species are “intermediate” between clustered and non-clustered organization (Figure 3D), and there is a gradation of both the percentage of piRNA loci in genes and the strength of the pausing-associated sequence signature downstream of the piRNAs (Figures 7H and 7I).

Overall, the aspects of piRNA biogenesis that we describe for *C. elegans* illustrate the utility of using a cross-species approach to investigate mechanistic questions in model organisms. We predict that this approach will be similarly important in addressing fundamental questions of mechanisms for other pathways of epigenetic regulation and genome defense, in part because these systems evolve rapidly across species.

## STAR★Methods

### Key Resources Table

**Table:** 

REAGENT or RESOURCE	SOURCE	IDENTIFIER
**Bacterial and Virus Strains**		

*E. coli* HB101	*Caenorhabditis* Genetics Centre	WormBase: HB101

**Chemicals, Peptides, and Recombinant Proteins**		

Trizol	Life Technologies	Cat# 15596018
Chloroform	Santa Cruz Biotechnology	Cat# sc-239527A
Isopropanol	Fisher Scientific UK Ltd	Cat# 15625890
UltraPure Buffer-Saturated Phenol	ThermoFisher Scientific	Cat# 15513039
Ethanol >99.8%	Sigma-Aldrich	Cat# 24103-1L-R
3M AcNa	VWR	Cat# E498-100ML
Glycogen	VWR	Cat# AC122
RPPH	NEB	Cat# M0356S
Quick dephosphorylation kit	NEB	Cat# M0508S

**Critical Commercial Assays**		

Illumina TruSeq Small RNA Library Prep kit	Illumina, Inc	Cat# RS-200-0012
D1000 TapeStation	Agilent Technologies	Cat# 5067-5582 and 5067-5602
Qubit	ThermoFisher Scientific	Cat# Q32850

**Deposited Data**		

All the data generated in this study has been deposited in the Sequence Read Archive (SRA), accession number SRP117954.	This study	Table S1

**Experimental Models: Organisms/Strains**		

List of *C. elegans* and other nematode strains.	This study	Table S2

**Oligonucleotides**		

List of oligonucleotides for CRISPR and genotyping.	This study	Table S3

**Software and Algorithms**		

Cutadapt v1.10	(Martin, 2011)	N/A
Bowtie v0.12	(Langmead et al., 2009)	N/A
Bowtie2 v2.2.9	(Langmead and Salzberg, 2012)	N/A
deeptools v3.1.2	(Ramírez et al., 2016)	N/A
Skewer v0.2.2	(Jiang et al., 2014)	N/A
kmc2	(Deorowicz et al., 2015)	N/A
SPAdes v3.5.1	(Bankevich et al., 2012)	N/A
Redundans v0.12	(Pryszcz and Gabaldón, 2016)	N/A
SOAPdenovo Gapcloser	(Luo et al., 2012)	N/A
RepeatModeler v1.0.8	(Smit and Hubley, 2008)	N/A
RepeatMasker v4.0.7	(Smit et al., 2013)	N/A
STAR v2.5.2	(Dobin et al., 2013)	N/A
BRAKER v1.9	(Hoff et al., 2016)	N/A
BLAST 2.2.28+	(Camacho et al., 2009)	N/A
Exonerate 2.2.0	EBI	https://www.ebi.ac.uk/about/vertebrate-genomics/software/exonerate
T-coffee	(Di Tommaso et al., 2011)	http://tcoffee.crg.cat
Trimal v1.4	(Capella-Gutiérrez et al., 2009)	N/A
RAxML v8.2.9	(Stamatakis, 2014)	N/A
MEME v4.10.1	(Bailey et al., 2009)	N/A
Infernal v1.1.2	(Nawrocki and Eddy, 2013)	N/A
Orthofinder v0.6.1	(Emms and Kelly, 2015)	N/A
TopHat v2.0.11	(Trapnell et al., 2009)	N/A
Bedtools v2.25.0	(Quinlan and Hall, 2010)	N/A
DEseq v1.30.0	(Anders and Huber, 2010)	N/A
Emboss dan v6.6.0	EBI	https://www.ebi.ac.uk/Tools/emboss/

### Contact for Reagent and Resource Sharing

Further information and requests for resources and reagents should be directed to and will be fulfilled by the Lead Contact, Peter Sarkies (psarkies@imperial.ac.uk).

### Experimental Model and Subject Details

Nematode strain details can be found in Table S2.

### Method Details

#### Nematode Culture and RNA Extraction

*C. elegans*, *Oscheius tipulae*, *Diploscapter pachys* and *P. pacificus* were grown at 20°C in standard nematode growth medium (NGM) agar plates feeding on HB101 *E. coli* seeded in LB medium. *P. oxycercus* was grown in the same way at 25°C. *P. sambesii* was grown at 25°C in low salt NGM feeding on HB101 seeded after resuspending in water. *H. bacteriophora* strain M31e was grown on lipid agar media using *Photorhabdus temperata* TRN16 as a food source. To enrich for germline tissue, *C. elegans*, *O. tipulae* and *H. bacteriophora* nematodes were synchronised by hypochlorite treatment and grown to adulthood. *P. sambesii* adults were selected using the COPAS sorter (Union Biometrica). RNA was prepared through Trizol-chloroform extraction and overnight isopropanol precipitation at -20°C.

#### Preparation of *P. oxycercus* Genomic DNA

Genomic DNA of *P. oxycercus* was prepared using a DNeasy Blood and Tissue kit (Qiagen), according to the manufacturer’s instructions. Library preparation and sequencing of *P. oxycercus* was performed by GATC Biotech.

#### Gonad Dissection and RNA Preparation

*C. elegans* and *P. pacificus* young adults gonads were obtained by dissecting the worms with a gauge needle in M9 medium and immediately transferring the gonadal arms to Trizol and RNA was extracted as described above. Multiple samples were pooled to obtain two final samples for each species with ∼500 ng germline total RNA. Whole-animal young adult worm samples were used as controls for germline enrichment.

#### Small RNA Library Preparation and Sequencing

RNAs were pretreated to modify 5’ ends in two ways. For RPPH treatment, 1-2 μg of total RNA was treated with 10 units (2 μl) RNA 5’ pyrophosphohydrolase (NEB) for 1 h at 37°C. The treated RNA was phenol-chloroform extracted and ethanol precipitated with sodium acetate and glycogen for 2 days, and resuspended in RNase-free water. For CIP-RPPH treatment, 3-5 μg of total RNA was treated with 4 μl of QuickCIP (Quick dephosphorylation kit, NEB) in a total volume of 40 μl for 90-120 min at 37°C. RNA was phenol-chloroform extracted, precipitated overnight with sodium acetate and glycogen and resuspended in RNase-free water.

Small RNA libraries from treated or untreated RNA were built using the TruSeq small RNA kit (Illumina) according to the manufacturer’s instructions except for an increase in the number of PCR cycles from 11 to 15. Libraries were eluted in 0.3 M NaCl, ethanol precipitated and quantitated with Qubit and TapeStation. Libraries were pooled in groups of 6 to 12 per lane and sequenced on an Illumina HiSeq2000.

The Illumina universal adapter was trimmed from small RNA reads using cutadapt v1.10 and reads were mapped to the corresponding genome assemblies with Bowtie v0.12 (Langmead et al., 2009) with parameters –v 0 –m 1.

#### Genome Assembly and Annotation of *P. oxycercus*

Quality control of *P. oxycercus* raw genomic data was assessed using Fastqc v0.10.1, and reads were quality trimmed using skewer v0.2.2 (Jiang et al., 2014) with parameters –q 30 –l 51. K-mer plots were generated with kmc, which revealed extensive heterozygosity. A preliminary single-end assembly was generated with Velvet (Zerbino and Birney, 2008) and contaminants were identified using blobtools (Kumar et al., 2013, Laetsch, 2016). The data were digitally normalised to 80x coverage to facilitate assembly. Assembly was carried out with SPAdes v3.5.1(Bankevich et al., 2012) correcting the reads with BayesHammer within the SPAdes pipeline. Assembly parameters were –k 21,33,55,77,99 –cov-cutoff auto --careful. *Plectus sambesii* was assembled as described in Rošić et al. (2018).

Examination of the resulting distribution of contig read coverages revealed a bimodal distribution suggesting heterozygosity. Haploid coverage contigs were collapsed and postprocessed with Redundans (Pryszcz and Gabaldón, 2016), that runs SSPACE3 and SOAPdenovo Gapcloser internally. RepeatModeler v1.0.8 (Smit and Hubley, 2008) was used to generate a species-specific repeat library that was concatenated with a nematode repeat library from RepBase. The combined library was used to mask the genome with RepeatMasker v4.0.7 (Smit et al., 2013).

To annotate the *P. oxycercus* genome, we generated rRNA-depleted paired-end total RNA-seq libraries. Quality control of the RNA-seq reads was performed with Fastqc and Skewer with parameters –q 30 –l 50. Reads were aligned to the assemblies with STAR v2.5.2 (Dobin et al., 2013) with default parameters except for --twopassMode Basic. Alignment BAM files were used for automated annotation using BRAKER v1.9 (Hoff et al., 2016).

#### Phylogenetic Profiling of piRNA Pathway Genes

Identification of Piwi from nematode genomes was performed essentially as described (Sarkies et al., 2015). Briefly, reciprocal blastp searches were performed on predicted protein sets using *C. elegans* proteins as a query sequence. In species where we did not identify a reciprocal best hit, (such as *C. plicata*), we additionally performed tblastn and exonerate searches against the genome sequence using the *C. elegans* protein as a query sequence, to verify the absence of the gene.

#### *De Novo* Motif Discovery and piRNA Annotation

Sequences 110 bp upstream and 30 bp downstream of 21U mapping sites were extracted and 10 subsets of 2000 sequences were randomly selected to predict motifs *de novo* using MEME v4.10.1 (Bailey et al., 2009) with parameters –dna –mod zoops –maxsize 2000000 -nmotifs 10. Genome-wide nucleotide content was used as a background model for MEME. The resulting motifs from each subset were compared to assess the consistency of the analyses, and the full dataset of upstream sequences was scanned with the obtained motif position weight matrix (PWM) using a custom Python implementation. For each species, the distribution of mapping positions showed a peak at the expected distance of 42 nt from the 21U mapping site. The distributions of PWM scores showed bimodal distributions, indicating good separation of true piRNA motifs from false positive hits. These distributions were used to define species-specific thresholds to select true positive motifs, which were annotated as piRNA loci. Motifs were used for genome-wide scans without 21U sequence information using the same threshold. In *H. bacteriophora*, upstream sequences displayed no positional bias for GTTTC nor any of the motifs predicted by MEME. We performed motif discovery on other small RNA lengths and 5’ nucleotides as controls, finding no enrichment of motifs. In the absence of small RNA sequencing data, *P. murrayi* piRNAs were identified through genome-wide motif scans using the *P. sambesii* motif, which provided good separation from false positives in the bimodal distributions of motif scores, due to high information content in the bipartite motif. Out of 3082 sites, we removed 39 corresponding to snRNA and other non-coding RNA genes annotated as described below.

To map early precursor transcripts, we mapped RPPH treated small RNA libraries to piRNA loci identifying reads containing 5’ and 3’ extensions to the 21U-RNA sequence as described previously (Weick et al., 2014).

#### Annotation of snRNA Genes and Prediction of SNAPc Motifs

We annotated SNAPc-dependent non-coding RNA loci across nematode genomes using Infernal v1.1.2 (Nawrocki and Eddy, 2013). RFAM alignments for U1, U2, U3, U4, U4atac, U5, U7, U11 and U12 snRNAs (Pol II-dependent), and U6 snRNA, U6atac snRNA, 7SK RNA and MRP RNA (Pol III-dependent) (see Supplementary tables for RFAM IDs) were used to build and calibrate Infernal covariance models with default parameters. Nematode genomes were searched with the trained models with default parameters. The 100 bp upstream of significant hits (E-value >1e-3) were extracted for motif prediction with MEME v4.10.2 (Bailey et al., 2009) with parameters –dna –mod zoops –nmotifs 10.

#### Identification and Analysis of SNPC-4 Motif-Only piRNAs in *C. elegans*

We regenerated the SNPC-4 binding motif using as input a set of sequences from germline-specific SNPC-4 binding sites defined in Kasper et al. (2014). We mapped 21U reads from existing small RNA sequencing libraries (Shi et al., 2013), extracted 100 bp of upstream sequence from 21U mapping sites, and performed scans using the regenerated SNPC-4 motif PWM. We defined a motif score threshold of 1500 based on the bimodality of the distribution of motif scores. We plotted the distribution of motif positions for the high-scoring subset and extracted the most abundant peak of sequences which corresponds to piRNA-like positioning of the SNPC-4 motif. We removed sites overlapping with snRNA and snoRNA sites defined by Infernal searches or present in WormBase annotations. We overlapped those sites with SNPC-4 ChIP-seq peaks defined in a *prde-1* mutant background (Kasper et al., 2014), with existing motif-independent piRNA annotations (Batista et al., 2008), and with WormBase TSS annotations, extending the TSSs 100 bp upstream and downstream. We estimated the expression of the 21U RNAs and potential precursors in N2, *prg-1* and *prde-1* mutants using data from Weick et al. (2014).

#### Phylogenetic Analysis of SNAPc Orthologs in Nematodes

Ortholog genes of human SNAPc subunits were identified via reciprocal BLAST analysis as described above. SNAPc ortholog sequences were aligned using T-Coffee (Di Tommaso et al., 2011), and the alignment was trimmed using Trimal v1.4 (Capella-Gutiérrez et al., 2009) with parameters -strict. The maximum-likelihood best tree was built with RAxML v8.2.9 using a PROTGAMMAGTR substitution model.

#### Estimation of Marginal Ancestral States of the SNAPc Motif in Nematodes

A guide tree was constructed using protein sequences from 1:1 orthologs defined with Orthofinder v0.6.1. Sequences were aligned with Clustal Omega v1.2, alignments were trimmed with Trimal v1.4 and concatenated into a supermatrix. Phylogenetic reconstruction was carried out using RAxML v8.2.9 using PROTGAMMAGTR as a substitution model. SNAPc sequence motifs for all species were converted into nucleotide sequences selecting the most probable base at each position. The alignment of the resulting sequences from all species was used as a input for estimation of ancestral states with RAxML with parameters -f A -t guideTree File -m GTRGAMMA -s AlignmentFile. In order to estimate the probability of a GTTTC being present in the ancestral SNAPc motif, the sites were assumed independent, thus the estimated probabilities of each nucleotide were multiplied together. The probability of a GTTTC having arisen independently in *P. sambesii* and *R. culicivorax* was estimated by multiplying the probability of a GTTTC occurring in both by the probability that GTTTC was not in the ancestral state i.e. 1-p(GTTTC ancestral)^∗^p(GTTTC *Plectus*)^∗^p(GTTTC *Romanomermis*).

#### piRNA Clustering Analysis and Comparison to the *C. elegans* Genome

We identified piRNA-enriched contigs by applying a binomial test against a null hypothesis of even distribution of piRNAs across the genome. We ordered the contigs by decreasing significance of enrichment, and a cumulative plot of piRNA counts by span was generated. This approach yielded a good separation between piRNA-enriched and piRNA-depleted contigs and allowed us to define the span covered by piRNAs for each species.

We validated this method using a fragmented *de novo* assembly of the *C. elegans* genome, where we successfully recovered the genomic regions corresponding to the *C. elegans* piRNA clusters. We applied the same method to the genome of *O. tipulae*, for which there is a good sequence-anchored linkage map. The top 10 most enriched contigs were found in two blocks mapping to chromosomes *Ot*IV and *Ot*V, consistent with the presence of clusters similar to *C. elegans*.

For each species, we defined gene orthology to *C. elegans* with Orthofinder v0.6.1(Emms and Kelly, 2015) running mcl 14.137. 1:1 orthologs were filtered from the final dataset (Supplementary tables, *all species-piRNA organisation*), and the *C. elegans* orthologs of genes localised in piRNA contigs for each species were identified. The density of those *C. elegans* orthologs across 100kb bins across the genome is shown in Figure 3B. *P. sambesii* and *P. murrayi* were not included in this analysis as the conservation of macrosynteny in these species is likely to be significantly lower than in the rest of species due to larger phylogenetic distance.

To quantify the extent of clustering, we calculated the cumulative frequency of total loci and identified the span required to reach 90% of the total loci. We then plotted this value against the total number of piRNAs to give the plot in Figure 3D. Multimodal regression analysis was performed using the Mixtools package in R.

#### Analysis of the Chromatin Environment of piRNAs

We used Integrative Genome Viewer to visualise *C. elegans* piRNA positions relative to genes and a variety of chromatin tracks. All analyses were done using *C. elegans* WS252 (ce11) as a reference. ChIP-seq tracks were generated using raw data from Janes et al. (2018). Reads were mapped to WS252 with bowtie2 with default parameters, multiple-mapping reads were removed, and the resulting alignments were normalised to 1x coverage and converted to bigwig tracks using deeptools v3.1.2 excluding read duplicates. Early embryo chromatin domain annotations (Evans et al., 2016) were lifted over to WS252 using liftover. *P. pacificus* chromatin tracks were generated using raw data from Werner et al. (2018) as described for *C. elegans* data. Chromatin domains were defined using ChromHMM chromatin states from Werner et al. (2018); states 2-3-4 were grouped as “active”, while 5-6-7 as “regulated”. piRNA locations in both species were intersected with defined chromatin domains using Bedtools v2.25.0 (Quinlan and Hall, 2010), and the significance of the enrichment relative to a uniform distribution of piRNAs across the genome was calculated with a Fisher’s Exact test.

#### Estimation of Chromatin Environment of piRNAs in Other Species

1:1 orthologs between *C. elegans* and each species were defined as described above, and annotated for chromatin state based on the state of the *C. elegans* ortholog. We tested the association of piRNA-containing genes with particular chromatin domain types with a Fisher’s exact test. We calculated the log_2_ of the odds ratio of a 2x2 table of genes with and without piRNA locus association, in active versus regulated chromatin.

We used *P. pacificus* data to benchmark chromatin domain predictions in a number of ways. We defined protein-coding genes to be in active or regulated domains if 75% of the gene locus was covered by active or regulated chromatin states as described above. We cross-compared the orthology-based predictions with these annotations.

We generated single-end RNA-seq reads, mapped them to their genome assemblies with Tophat (Trapnell et al., 2009) with parameters -i 30 -I 20000, and recovered read counts per gene with htseq-count with default parameters. Given than gonad expression distributions in RPKM showed clear bimodality in both species, we used k-means clustering to estimate an expression threshold to classify genes in high and low expression groups. We cross-compared expression groups with annotated chromatin domains in *C. elegans* and with orthology-based predictions in *P. pacificus*. Both species showed similar correspondence between expression groups and chromatin domain groups.

#### Analysis of piRNA Organization Relative to Protein-Coding Genes

The localisation of piRNAs relative to genes and intronic regions in each genome was calculated using Bedtools intersect v2.25.0. We randomly simulated piRNA positions genome-wide to generate null distributions, and simulated piRNA positions within their contig of origin to account for potential regional biases in those genomes. We defined a “genicness score” for each species as the genic/intergenic ratio of piRNA locus positioning in the real dataset divided by the median of the 100 genic/intergenic ratios of simulated datasets (equivalent to a log_2_ odds ratio). For promoter association of piRNAs, we defined promoter regions as regions of 200 bp, 500 bp or 1 kb upstream of the first exon of protein-coding genes, and calculated piRNA enrichment relative to non-promoter intergenic regions, for all promoters, and for active/regulated promoters defined by orthology as described above.

#### Expression of Artificial piRNA Loci

We used CRISPR-Cas9 to insert an artificial piRNA locus with a 21U-RNA sequence absent from the *C. elegans* genome. We based the artificial piRNA on the endogenous piRNA 21UR-3922 (IV:15671231-15671251) and edited the mature piRNA sequence such that it had no match to the *C. elegans* genome (TCGGATCGGGTCATACCGGAT > TCGGATCGGGTCATACGCGAT), leaving the upstream and downstream parts the same (mature 21-RNA sequence in capital letters):

aaaaaattttgtaatgtttcacatttaccataaaattgtcctaatttaaaactgaattgaTCGGATCGGGTCATACGCGATttaaaacattaaatgtgtat

This construct was inserted into two regulated domains (IV:5583983, IV: 14055156), or into two active domains (IV:5391471, IV: 14070892) by injecting preformed Cas9-gRNA complex along with the appropriate repair oligo (Table S2), with *dpy-10* as a coinjection marker (Paix et al., 2015). Correct insertions were verified by PCR and Sanger sequencing (see Supplementary tables for CRISPR and genotyping oligonucleotides).

We modified the region immediately downstream of the mature piRNA sequence to increase its GC content, and thus the melting temperature of the region, and inserted this new locus (21UR-3922m “bad terminator”) into the one of the regulated domain locations (IV:5583983).

Two strains carrying each insertion representing independent insertion events were generated. The strains were synchronised by hypochlorite treatment and grown from L1 for 72 h for RNA isolation, and RPPH libraries were prepared as described above. We recovered 21U-RNA and piRNA precursor counts for each piRNA locus. We used DEseq2 to estimate library size factors and identify differentially expressed 21U-RNAs. Additionally, we normalised the mature piRNA and precursor read counts of the artificial locus relative to those of the endogenous locus.

#### Generation of a mes-2 Deletion Using CRISPR-Cas9

The mes-2(we28) allele, which deletes 4298 bp of mes-2 gene (II: 14,383,918 – 14,388,215, ce10), was generated using CRISPR-Cas9 genome editing as in (Paix et al., 2015). tracrRNA, crRNAs and the ssODN repair template were purchased from IDT (see supplementary tables for sequences). mes-2(we28) was balanced over a mnc1[dpy-10(e128) unc-52(e444) umnls32] balancer marked with myo-2::GFP to create strain JA1805.

#### Analysis of piRNA Expression in *mes-2 C. elegans* Worms

Small RNA sequencing data was processed as described above. Library size factors used to normalise piRNA counts were estimated with DESeq2 using miRNA counts. We additionally normalised the data using total miRNA reads and miR-35 reads to estimate size factors, all of which were consistent with a decrease in piRNA content in libraries from *mes-2(we28)* homozygous worms relative to heterozygous siblings. Precursor reads were selected as reads >22 nt mapping 2 nt upstream of mature 21U-RNA sites. Library size factors for short-capped RNAs were estimated by recovering the number of reads mapping to annotated wormbase TSSs (Chen et al., 2013).

#### Effect of SNPs on piRNA Motif Scores

We downloaded genomic variation data for *C. elegans* from the *C. elegans* natural diversity resource (CeNDR, https://www.elegansvariation.org/, Cook et al., 2017). *C. briggsae* SNPs were taken from (Thomas et al., 2015). *P. pacificus* SNPs were downloaded from the *Pristionchus* variome site (http://www.pristionchus.org/variome/, Rödelsperger et al., 2014). To establish the impact of SNPs on piRNA motifs, we identified Ruby motifs using the specific position weight matrices (PWM) and selected motifs with a score >=500 log odds ratio and recalculated the motif scores after substituting any SNPs. For piRNA loci with a log odds ratio >1000, we identified SNPs with a predicted effect of >=400 change in log odds score (*C. elegans*, *C. briggsae*) or >=200 for *P. pacificus* (due to lower motif information content). We determined whether SNPs affecting piRNAs were major alleles (present in >90% of strains) or minor alleles (present in <10% of strains). We explored their association with annotated piRNA clusters, chromatin domains and genes using Bedtools v2.25.0 for data integration.

#### Calculation of Tm Profiles Around piRNA Transcription Start Sites

Sequences 200 bp upstream and downstream of piRNA motifs were extracted using the Bedtools module getfasta v2.25.0, and predicted DNA-DNA melting temperature (T_m_) profiles were calculated using EMBOSS dan v6.6.0 with a window size of 9 nucleotides, a window shift of 1 nucleotide and default DNA and salt concentrations. Stratification of piRNA loci was carried out by calculating the strength of the T_m_ profile as the positive difference of T_m_ values to background at the high T_m_ region (TSS to +19), plus the negative difference of T_m_ values to background at the low T_m_ region (+20 to +40). Background was defined as the average T_m_ at -200 to -100 and +100 to +200 from the TSS.

#### Comparison of Tm Profiles across Species

Quantification of T_m_ profiles across genomes is challenging given the differences in GC content across genomes that result in differences in background nucleotide composition around piRNAs. We calculated this metric in two ways: first, we used a Bayesian Integral Log-Odds approach (BILD) (Yu et al., 2015), using the genome-wide nucleotide composition as background model. This analysis gives a measure of entropy of the sequence at each position, and captures the piRNA termination region as a peak in entropy. We calculated the strength of termination as the area of this peak. Second, we calculated the difference between the maximum T_m_ value of the high GC content region and the minimum T_m_ value of the low GC content region. Notably, we found no correlation between genomic GC content and the strength of pausing signals.

#### Analysis of piRNA Expression in Low and High Tm Loci

To compare expression of piRNAs from loci with different pausing signature strengths we used two independent methods to normalize library size. We first normalised piRNA counts to total non-structural mapped reads (non-rRNA and non-tRNA). Second, we fitted a linear model to miRNA counts across pairs of libraries, and inferred size factors as the estimated value of the slope +/- 1.96 times the standard error of the estimate.

#### Analysis of piRNA Precursors

For analysis of piRNA precursors, CIP-Rpph reads (this study) or nuclear short capped RNA-seq reads (Weick et al., 2014) with 5’ ends mapping exactly 2 nt upstream of annotated 21Us were selected. We tested the difference in piRNA precursor length at CIP-RPPH libraries by a Wilcoxon rank sum test.

We used the length distributions of rRNA degradation products (reads mapping sense to rRNA) as a control to rule out variability across libraries due to size selection during library preparation. To compare the lengths of N precursors from library 1 and M precursors from library 2, we sampled N and M rRNA structural reads longer than 23 bases from each library, and calculated a Wilcoxon rank sum test p-value. We repeated the procedure 1000 times to calculate a null distribution of p-values. We noticed that differences in sequencing depth can bias the length distribution of unique piRNA precursor sequences, since longer precursors tend to be less abundant. To control for this, we generated 10000 subsamples of 2500 precursor sequences from N2-TFIIS library pairs (4 biological replicates), by weighted sampling of piRNA precursors according to their abundance (number of reads). We generated a distribution of p-values by a one-tailed Wilcoxon rank sum test (TFIIS>N2) and compared this distribution to a null distribution of p-values generated by comparing pairs of subsets of precursors sampled from the N2 library for each biological replicate. We analysed the parn-1 (tm869) mutant data as described above, with exception of a sample size increase to 5000 piRNA precursor sequences, due to increased sequencing depth in these samples resulting in the detection of a higher number of unique precursor sequences.

#### Analysis of 3′ Cleavage Products

For analysis of TFIIS-derived 3’ cleavage products fragments, all 21U reads were removed to avoid any potential interference from 21U reads coming from annotated and unannotated piRNA loci. Uniquely mapping reads longer than 10 bases mapping at positions +28 to +38 to the TSS were counted in each replicate and normalised to total non-structural mapped reads. The total number of unique sequences was quantified and normalised to the total number of unique non-structural mapped reads.

### Quantification and Statistical Analysis

Statistical analyses and quantifications have been described in the relevant STAR Methods sections above.

### Data and Software Availability

Sequencing data has been submitted to the SRA Study SRP117954: Evolution of piRNAs in nematodes. The *P. oxycercus* and *P. sambesii* genomes have been uploaded to http://caenorhabditis.org/.

## Supplemental Information

Document S1. Figures S1–S11

Table S1. Data Sources, Related to Figure 1 Contains the genome builds used for all analyses and the sources of high-throughput sequencing data analyzed in this study, either previously published or deposited as part of the current work.

Table S2. Nematode Strains, Related to Figure 1 Contains strains obtained for this study from available sources.

Table S3. Oligonucleotides, Related to Figures 4 and 6

Table S4. piRNA Information, Related to Figures 4 and 6 (A) piRNA organization across nematode genomes, relates to Figure 4. (B) piRNAs and chromatin domains in *C. elegans* and *P. pacificus*, relates to Figure 4. (C) Mature piRNA abundance and melting temperature profile scores at piRNA loci, relates to Figure 6.

Data S1. piRNA Annotations in Nematodes, Related to Figure 1

Data S2. Evolution of SNPC-4 Orthologs and snRNA Promoter Motifs in Nematodes, Related to Figure 2 (A) An extended tree with the SNAPc binding motifs across nematodes, along with the predicted ancestral state at key nodes. (B) The presence/absence of human SNAPc complex members in nematodes. (C) A phylogenetic tree of SNPC-4 orthologues across nematodes, with *D. melanogaster* and *H. sapiens* SNPC-4 proteins included for comparison. (D) An overview of the alignment of SNPC-4 orthologues with key domains and regions highlighted. D shows alignments of the Myb domains in SNPC-4.

Document S2. Article plus Supplemental Information
