# Supplementary material for: Comparative Epigenomics Reveals that RNA Polymerase II Pausing and Chromatin Domain Organization Control Nematode piRNA Biogenesis
Source: Dev Cell. 2019 Mar 25;48(6):793–810.e6. doi: 10.1016/j.devcel.2018.12.026 (PMC6436959; doi:10.1016/j.devcel.2018.12.026)
Supplement: Document S1. Figures S1–S11 [file mmc1.pdf]

Developmental Cell, Volume *48*

Supplemental Information

Comparative Epigenomics Reveals that RNA Polymerase II Pausing and Chromatin Domain Organization Control Nematode piRNA Biogenesis

Toni Beltran, Consuelo Barroso, Timothy Y. Birkle, Lewis Stevens, Hillel T. Schwartz, Paul W. Sternberg, Hélène Fradin, Kristin Gunsalus, Fabio Piano, Garima Sharma, Chiara Cerrato, Julie Ahringer, Enrique Martínez-Pérez, Mark Blaxter, and Peter Sarkies

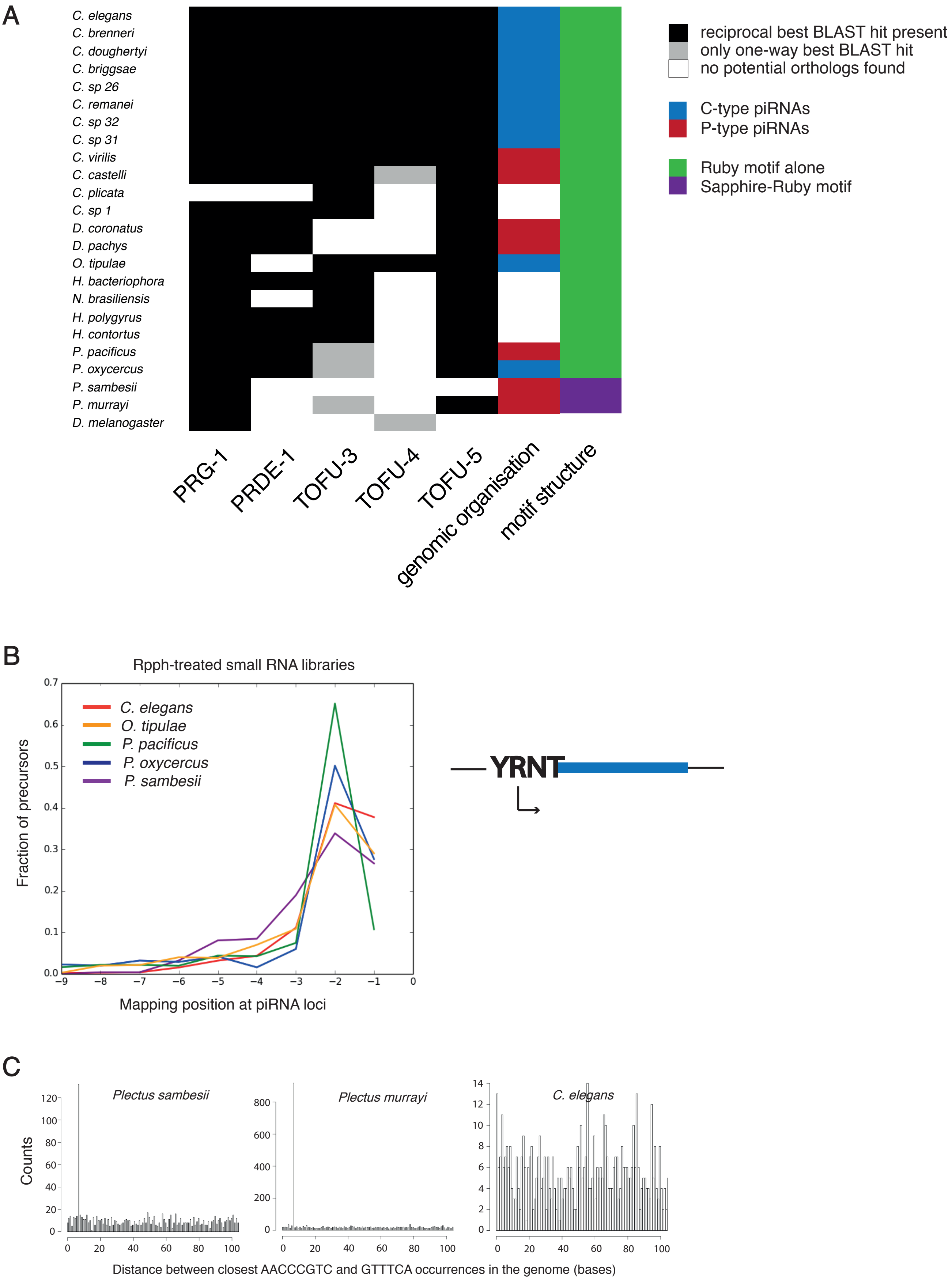

Figure S1 (relates to Figure 1)

Figure S1. Further analysis of piRNA transcription (relates to Figure 1)

A. Presence/absence of piRNA pathway gene orthologs across nematodes.

B. Transcription initiation at piRNA loci relative to mature 21U sequences across nematodes. 5' extensions of mapped piRNA precursor transcripts are shown.

C. Co-occurrence of GTTTC and AACCCGTC motifs in *Plectus sambesii* and *Plectus murrayi* but not in *C. elegans*.

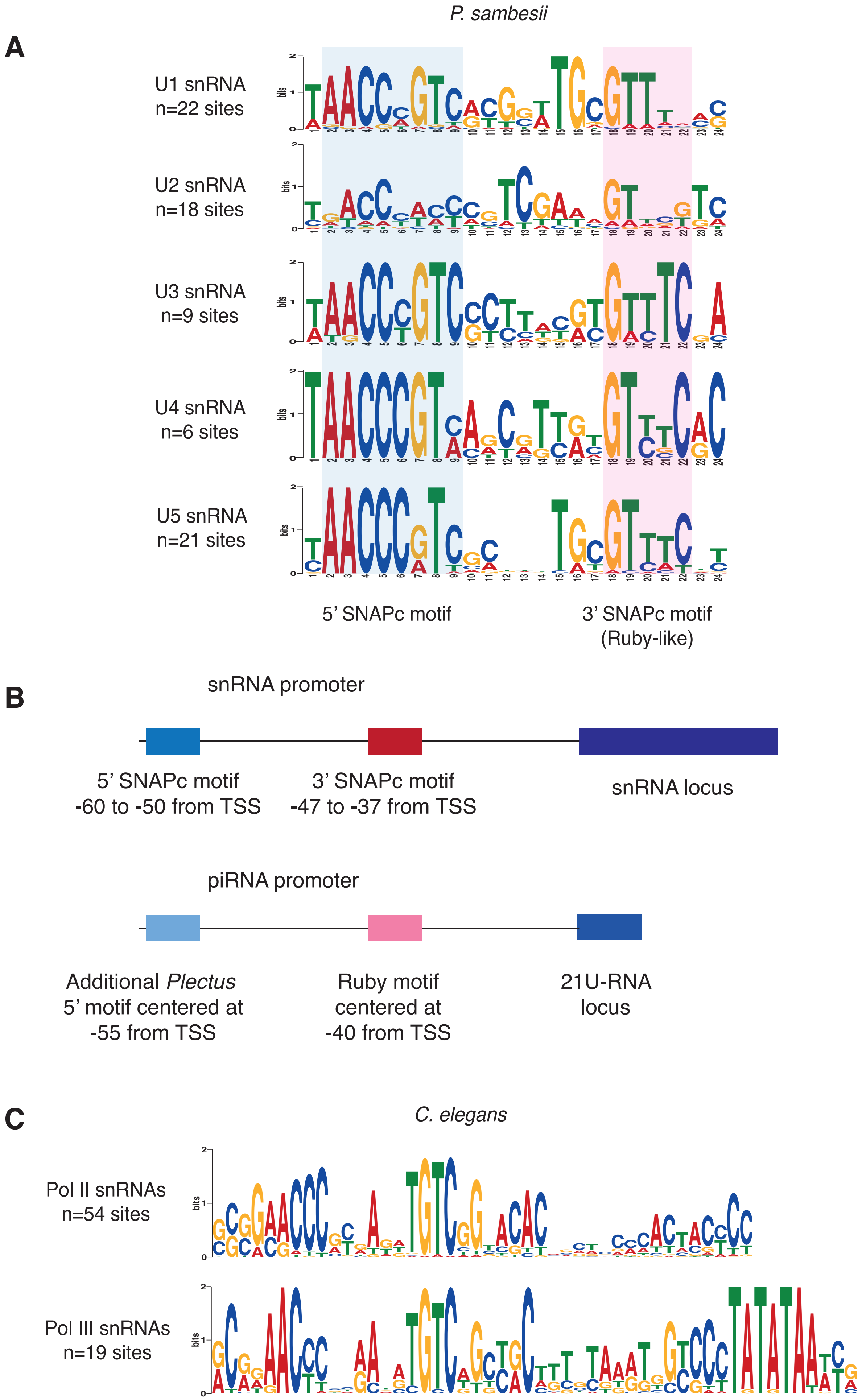

Figure S2 (relates to Figure 2)

Figure S2. snRNA promoter motifs in *P. sambesii* and *C. elegans* (Relates to Figure 2)

A. Upstream motifs in several classes of Pol II snRNAs in *P. sambesii*.

B. Description of sequence elements found in *P. sambesii* snRNA and piRNA promoters.

C. Upstream motifs in Pol II and Pol III snRNA promoters in *C. elegans*, matching SNPC-4 binding motifs predicted by ChIP-seq (Kasper et. al., 2014).

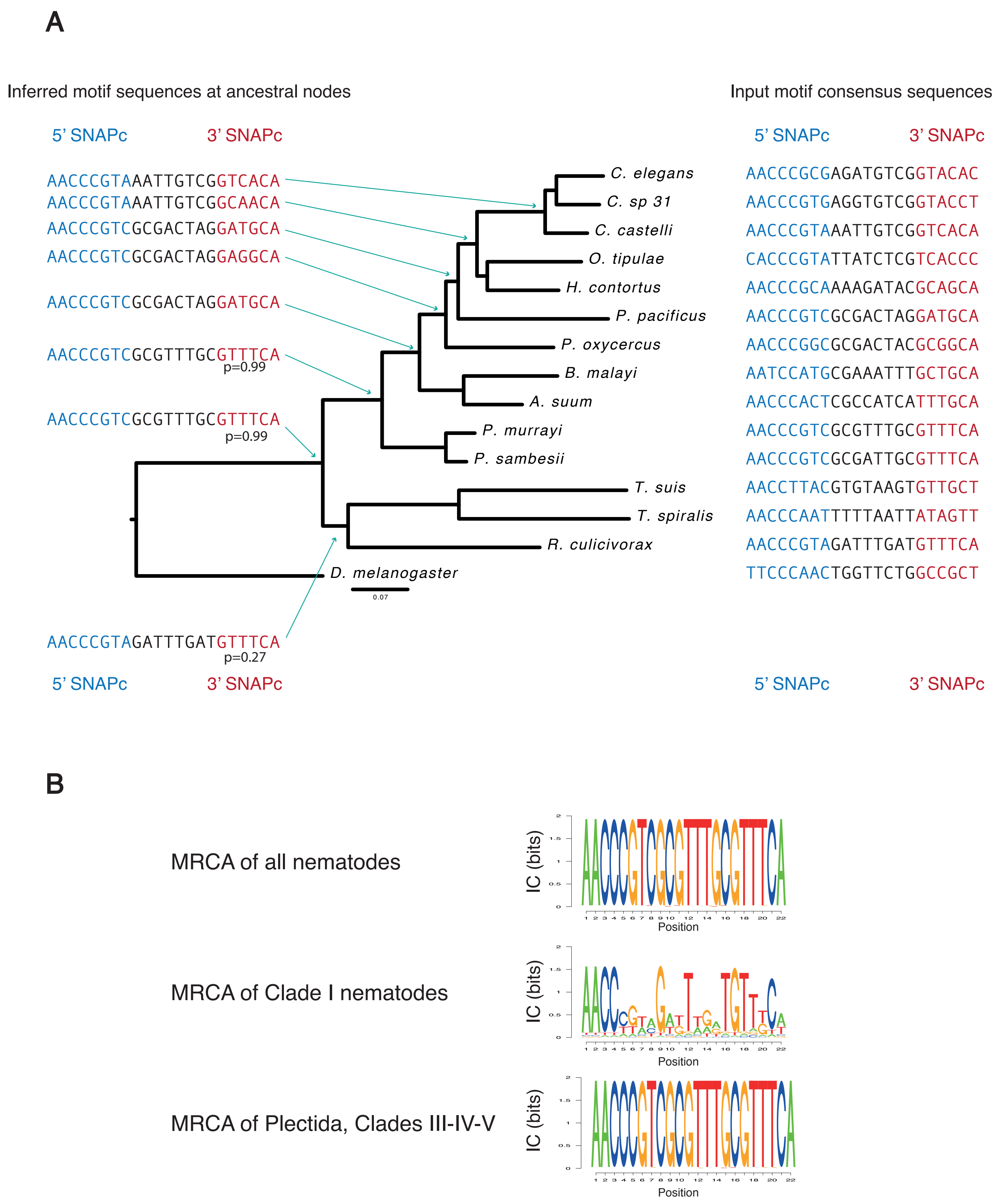

Figure S3- relates to Figure 2

Figure S3. The ancestral nematode SNAPc motif likely contained Ruby motif-like sequences in its 3' half (Relates to Figure 2)

A. Marginal ancestral states of SNAPc motif sequences in the nematode phylogeny. The guide tree was constructed using 1:1 ortholog protein sequences, and used as a reference to derive ancestral state estimates using motif sequences from extant taxa. The estimated p-value for the presence of the GTTTC in the 3' half of the motif is displayed at the relevant nodes.

B. Sequence logo representation of the marginal ancestral probabilities for selected ancestral nodes.

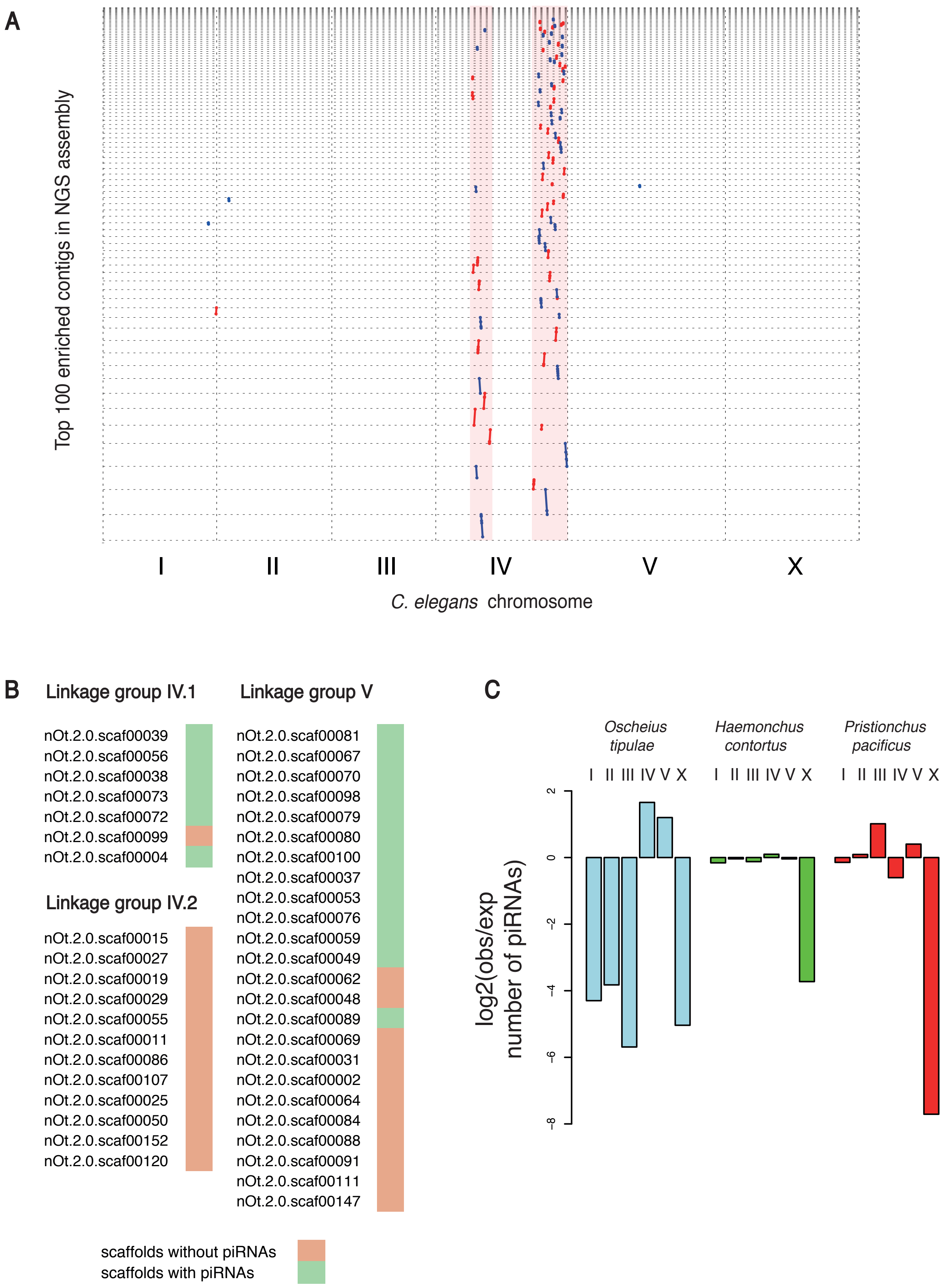

Figure S4- relates to Figure 3

Figure S4. Validation of piRNA clustering analysis methods (Relates to Figure 3)

A. Dot plot showing the alignment of the top 100 contigs enriched in piRNAs in a short-read *C. elegans* genome assembly (see Data S1, *Data sources*) to the *C. elegans* reference genome. These map exclusively to *C. elegans* piRNA clusters in IV.

B. Identification of scaffolds enriched in piRNAs in the *Oscheius tipulae* linkage map.

C. Chromosomal enrichment of piRNAs in *O. tipulae, P. pacificus and H. contortus.*

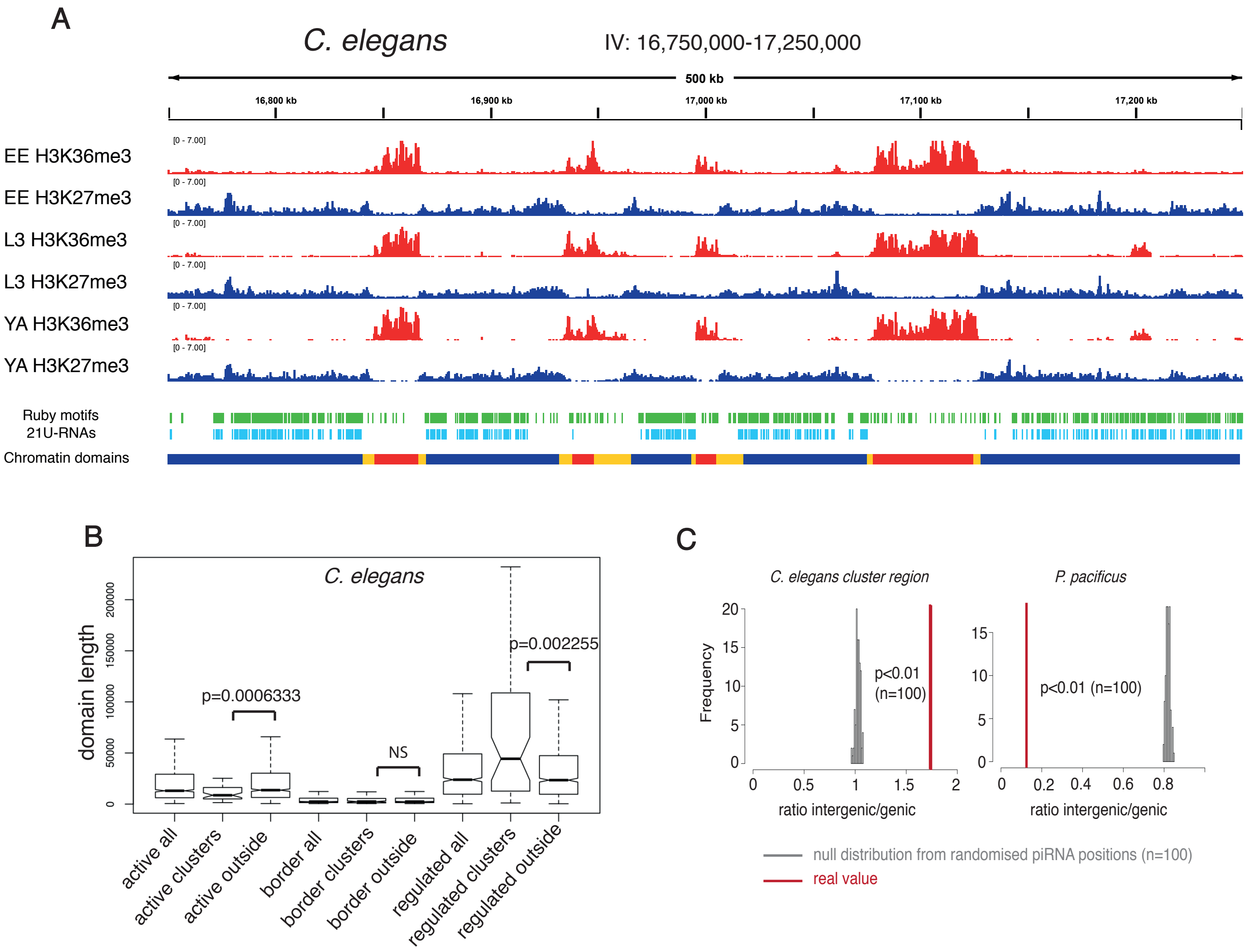

D

		Active	Regulated	
C. elegans	**within clusters**			(fold enrichment) (fold depletion)
	piRNA loci (motif+21U)	4 (286.05)	9138 (1.20)	
	scanned Ruby motifs	314 (5.16)	12416 (1.16)	**57.77**-fold more likely in regulated chromatin
	% motifs expressed	1.27	73.60	
	**outside clusters**			
	piRNA loci (motif+21U)	57 (1.73)	289 (1.95)	
	scanned Ruby motifs	12834 (1.14)	15309 (1.11)	**4.25**-fold more likely in regulated chromatin
	% motifs expressed	0.44	1.88	
P. pacificus	piRNA loci (motif+21U)	27206	3758	**10.6**-fold more likely in active chromatin
	scanned Ruby motifs	135710	198087	
	% motifs expressed	20.04	1.98	

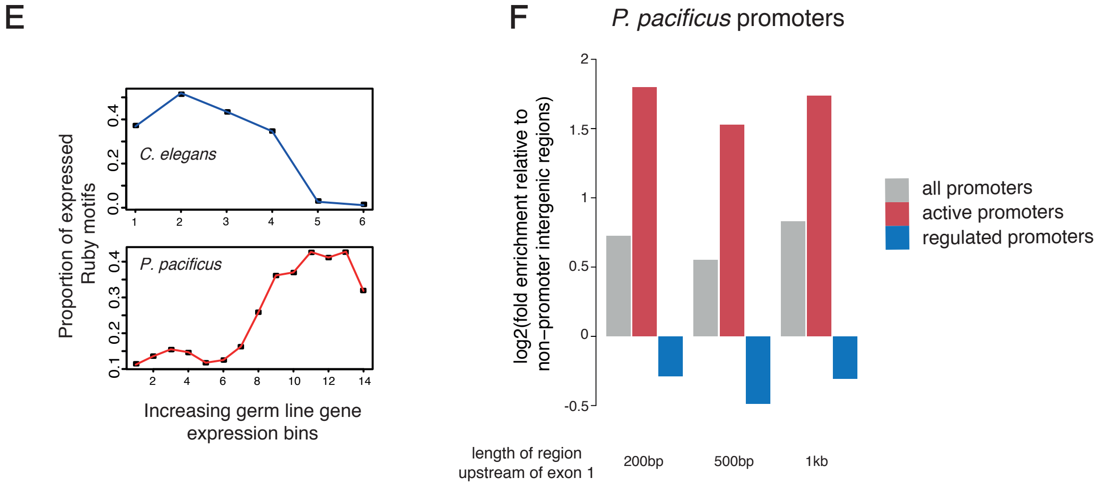

Figure S5- relates to Figure 4

Figure S5. Regulation of piRNA biogenesis by the chromatin environment (Relates to Figure 4)

A. Examples of H3K27me3 and H3K36me3 profiles at piRNA cluster regions in the early embryo, L3 and young adult stages.

B. Size of regulated and active domains within *C. elegans* piRNA regions compared to the rest of the genome.

C. Comparison of intergenic/genic piRNA proportions to simulated positions (n=100 random simulations).

D. Summary table of annotated piRNA loci and scanned Ruby motifs and their chromatin domain localisations in *C. elegans* and *P. pacificus*.

E. Proportion of genic scanned Ruby motifs that are expressed into 21U-RNAs in increasing bins of host protein-coding gene expression in *C. elegans* and *P. pacificus*.

F. Enrichment of piRNA loci in active and regulated promoter regions relative to non-promoter intergenic regions in *P. pacificus*. Promoters were defined as 200bp, 500bp and 1kb regions upstream of exon 1.

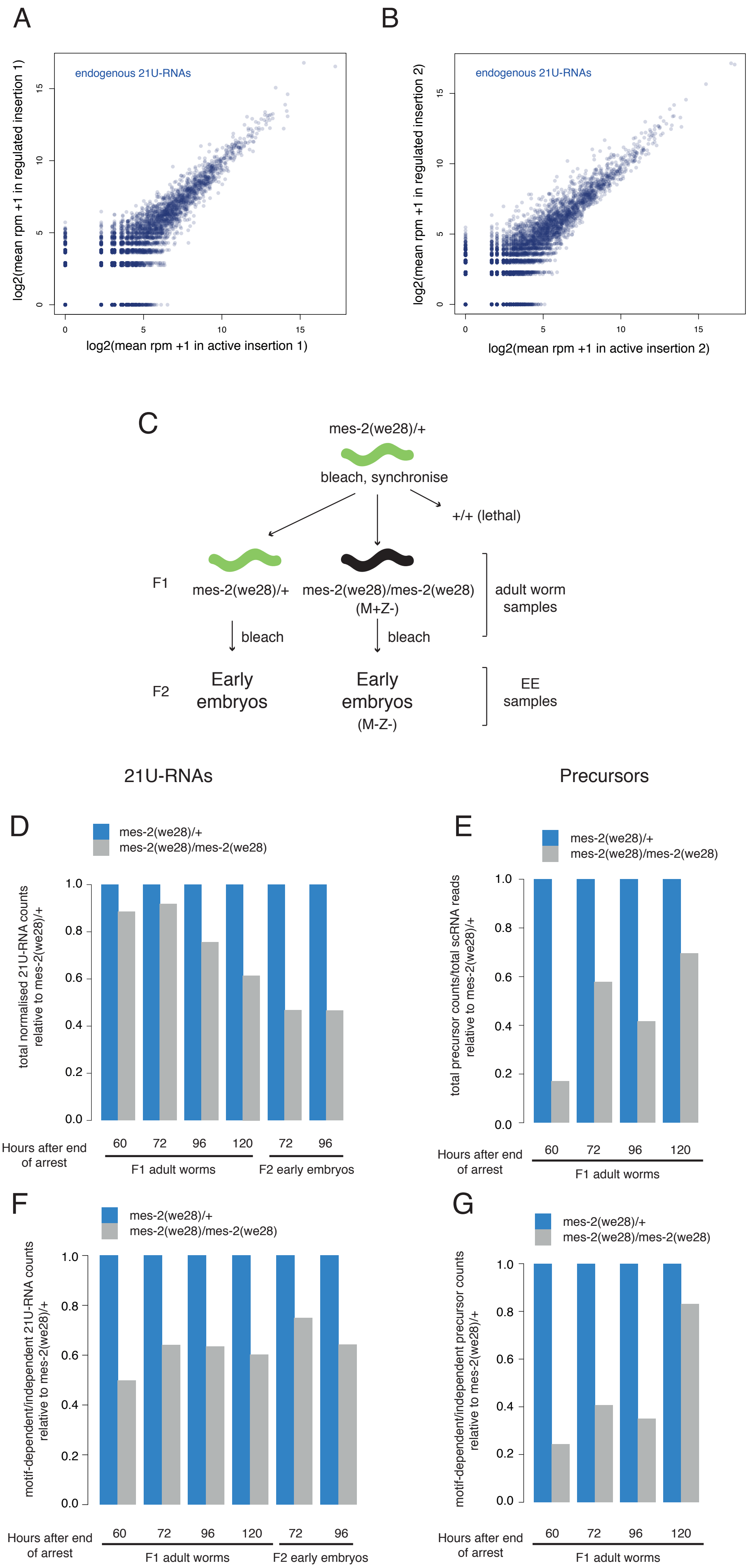

Figure S6- relates to Figure 4

Figure S6. MES-2 dependent H3K27me3 chromatin domains are important for piRNA expression (Relates to Figure 4)

A-B. Endogenous piRNA expression is not affected in artificial piRNA insertion strains. Comparison of log2(reads per million of piRNA reads) between active and regulated insertion strains.

C. Experimental design to assess piRNA expression in *mes-2(we28)* mutants.

D. Total normalised 21U-RNA reads in *mes-2(we28)* mutants relative to heterozygous siblings.

E. Total normalised piRNA precursor reads in *mes-2(we28)* mutants relative to heterozygous siblings.

F. Ratios of motif-dependent to motif-independent 21U-RNA total reads in *mes-2(we28)* mutants relative to heterozygous siblings.

G. Ratios of motif-dependent to motif-independent piRNA precursor total reads in *mes-2(we28)* mutants relative to heterozygous siblings.

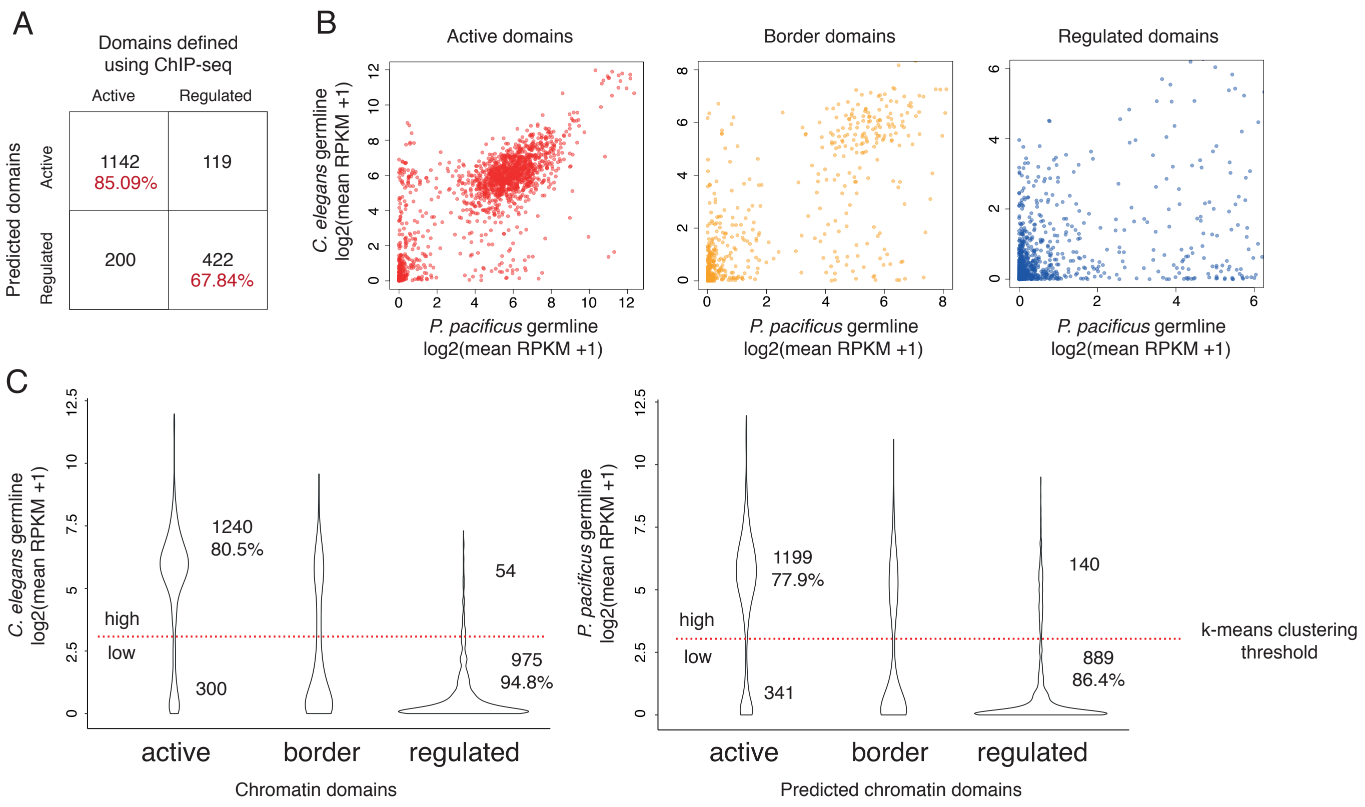

Figure S7- relates to Figure 4

Figure S7. Validation of chromatin domain predictions in *P. pacificus* (Relates to Figure 4)

A. Comparison between predicted and ChIP-seq-defined chromatin domain locations of protein-coding genes in *P. pacificus*.

B. Germline expression of 1:1 ortholog pairs in *C. elegans* and *P. pacificus* stratified by their *C. elegans* chromatin domain assignations.

C. The germline expression levels of *C. elegans* and *P. pacificus* genes are consistent with their assigned chromatin environment. The red line represents a k-means clustering threshold defining groups of genes with high and low expression. The number of genes in the high and low expression groups are shown. The correspondence of expression levels with chromatin domains is similar in both species.

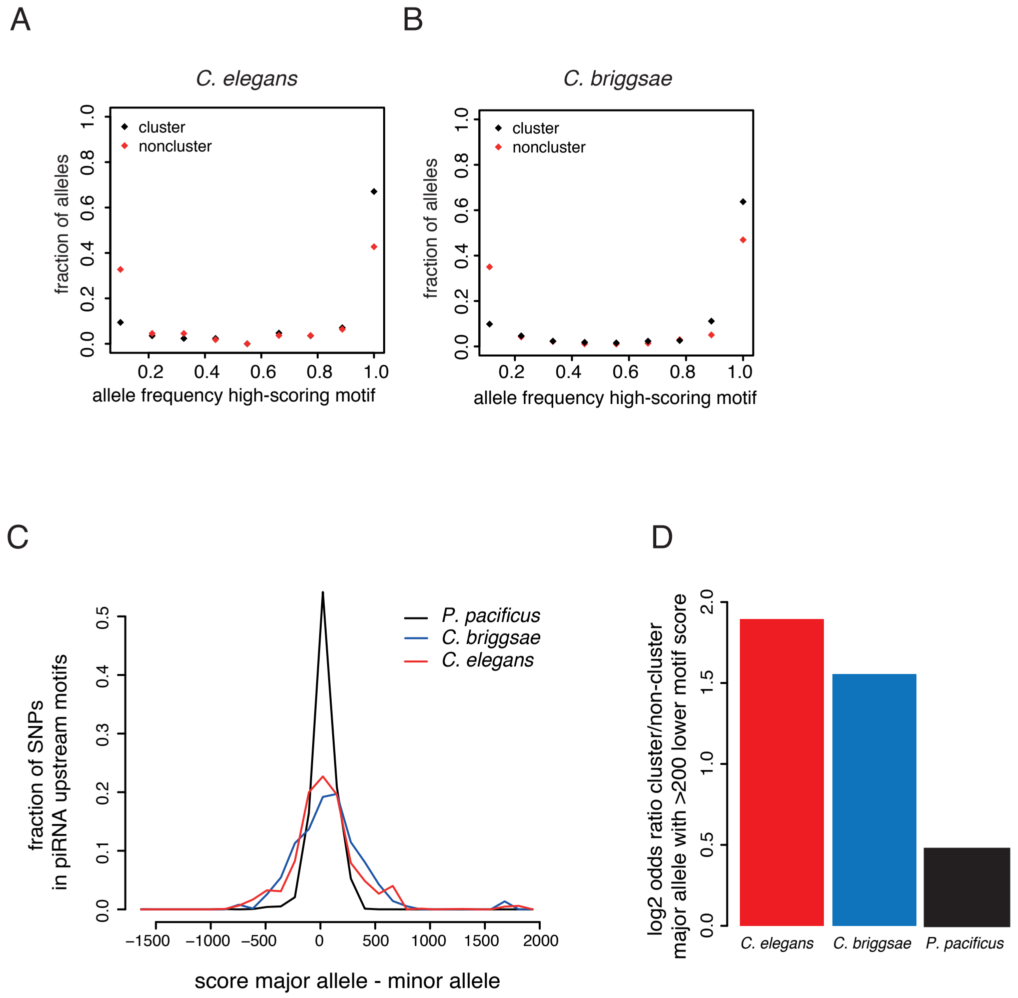

Figure S8- relates to Figure 5

Figure S8. piRNA loci are under selection in both C-type and P-type species (Relates to Figure 5)

A-B. Allele frequency distribution of SNPs predicted to alter piRNA motif strength by >400 in *C. briggsae* and *C. elegans*.

C. Distribution of the predicted changes in motif scores as a result of all SNPs mapping to piRNA loci in *C. briggsae*, *C. elegans* and *P. pacificus*.

D. Enrichment of high scoring motifs within piRNA clusters in *C. elegans* and *C. briggsae* using the lower threshold of a >200 change in strength, compared to the difference observed between the 10 highest density contigs and the 10 lowest density contigs in *P. pacificus*.

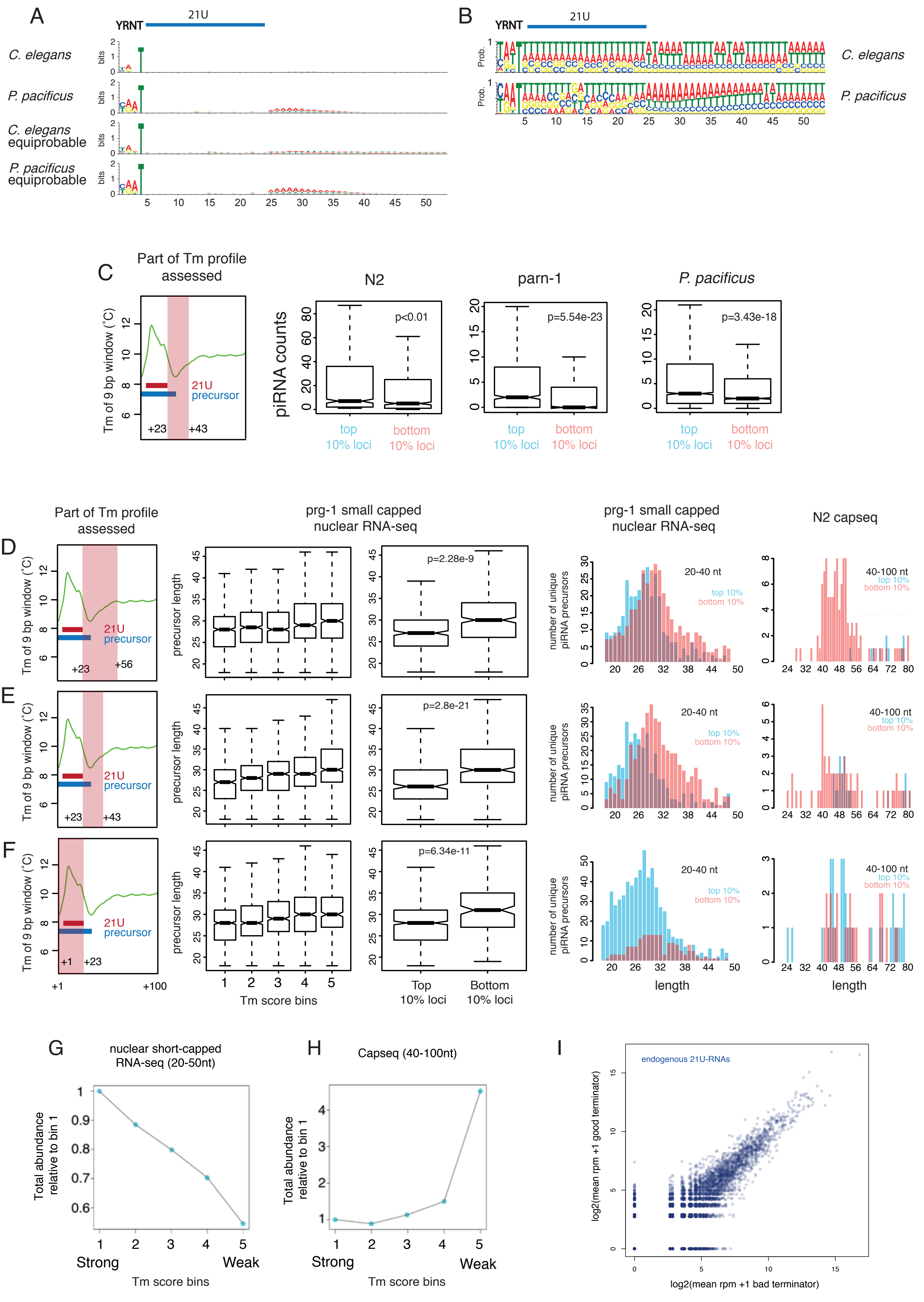

Figure S9- relates to Figure 6

Figure S9. Pol II pausing signals are important for piRNA biogenesis (Relates to Figure 6)

A. Entropy sequence logos of piRNA loci in *C. elegans* and *P. pacificus* using either genomic GC content or equiprobable nucleotide frequency as the background model.

B. Nucleotide frequency logos of piRNA loci in *C. elegans* and *P. pacificus*.

C. Effect of the pausing signal downstream of the 21U sequence on piRNA abundance.

D-F. Dissection of the effect of different parts of the pausing-associated sequence signal on piRNA precursor length and abundance.

G-H. Quantification of total piRNA precursor abundance across bins of decreasing strength of the pausing-associated sequence signal.

I. Endogenous piRNA expression is not affected in the bad terminator artificial piRNA insertion strains. Comparison of log2(reads per million of piRNA reads) between good and bad terminator insertion strains.

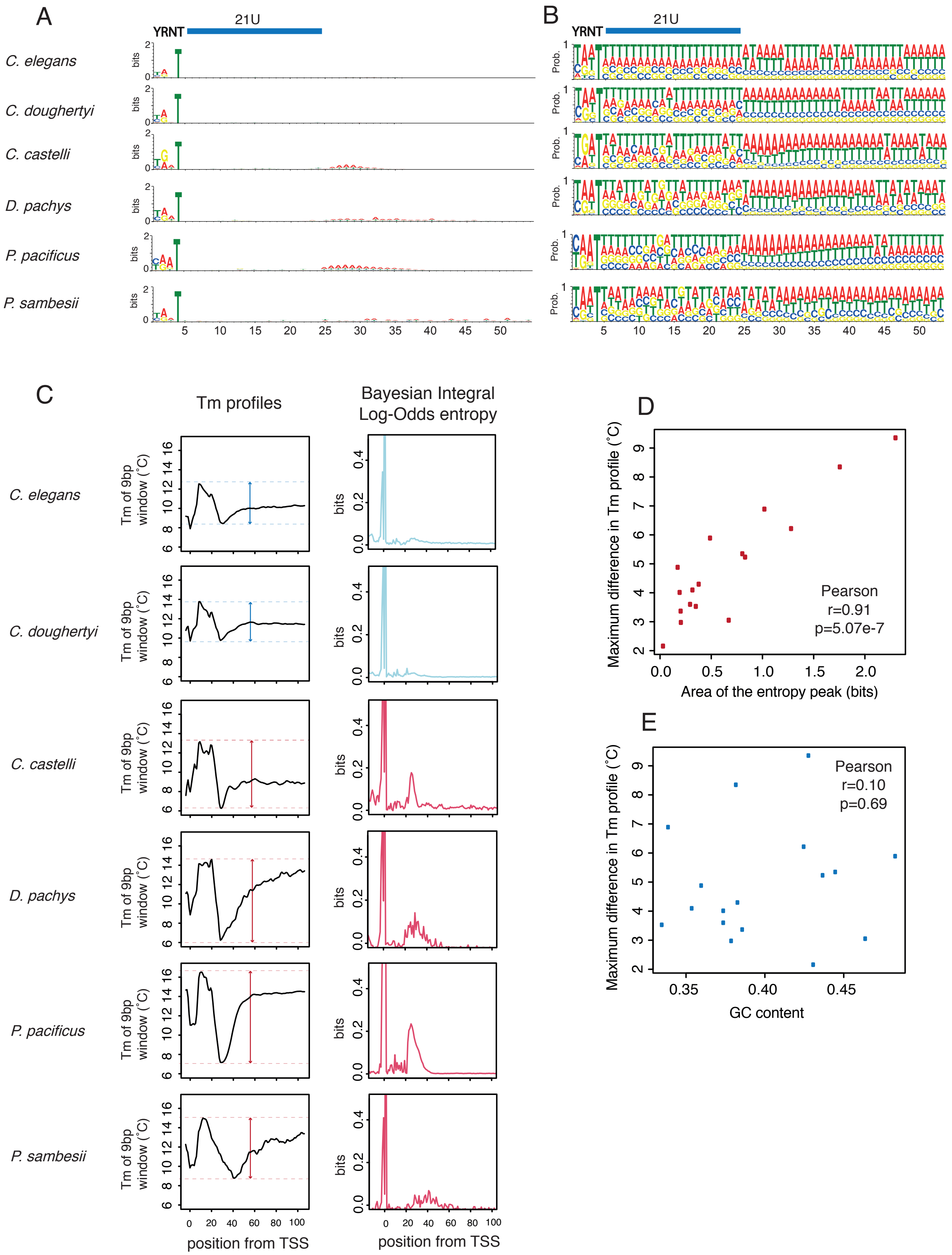

Figure S10- relates to Figure 6

Figure S10. Quantification of the strength of Pol II pausing signals across nematodes (Relates to Figures 6 and 7)

A-B. Sequence logos downstream of piRNA loci in several nematodes.
C. Melting temperature profiles and Bayesian Integral Log-Odds (BILD) entropy of the sequence around piRNA loci across nematodes. The strength of the pausing signature was calculated as the difference between the maximum value of the high GC content peak and the lowest value of the high AT content region from the Tm profiles, and alternatively as the area under the peak downstream of the 21U relative to background from the entropy profiles.
D. Relationship between the different measures of the strength of pausing signals (Spearman $r > 0.9$).
E. Relationship between genomic GC content and the measures of the strength of pausing signals (Spearman $r < 0.1$).

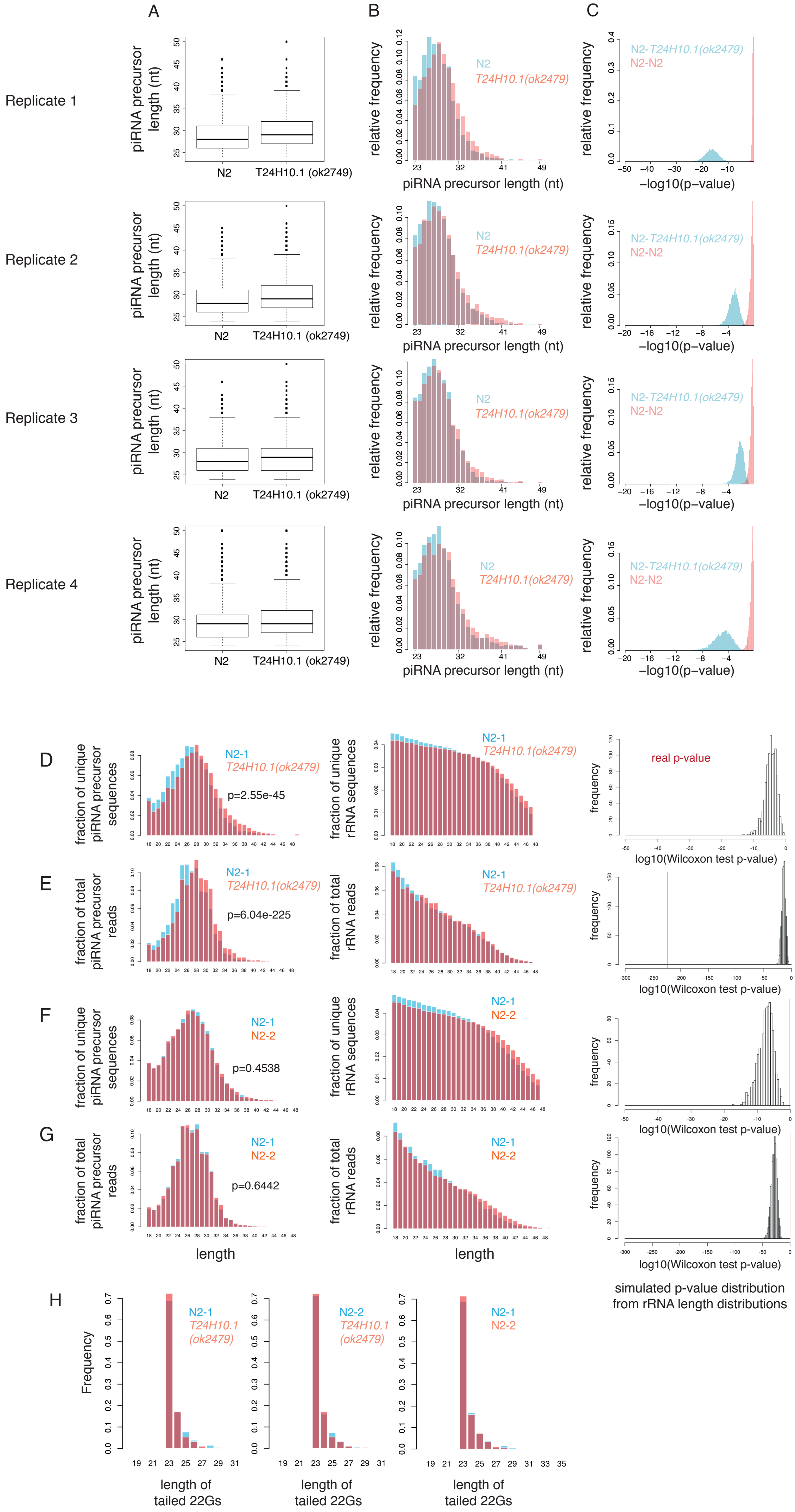

Figure S11- relates to figure 7

Figure S11. Loss of function of TFIIS modulates piRNA biogenesis in *C. elegans* (Relates to Figure 7)

A-B. Length distribution of piRNA precursors in subsets of 2500 precursors sampled according to relative abundance in N2 and tfiis(ok2749) mutants.

C. P-value distributions of a Wilcoxon rank sum test for a difference in length, in 10,000 N2 vs TFIIS random subsets (blue) and N2-N2 random subsets (pink).

D-G. Comparison of unique and total piRNA precursor read length distributions in N2 and TFIIS mutant *C. elegans*. The length distributions of total and unique rRNA degradation fragment reads were plotted as a control. P-values for the increase in length were calculated using a Wilcoxon rank sum test. P-values were compared against a null distribution of p-values calculated by sampling sets of a matching number of unique or total reads from the rRNA length distributions (sampling reads >23 nt). In TFIIS-N2 comparisons, the real p-value is much lower than the calculated null distribution, while in comparisons between two independent N2 libraries, the real p-value is greater, showing that the differences in precursor length are not a result of a global shift in read length in the libraries due to technical variation.

H. Length distribution of tailed 22G-RNAs in N2 and TFIIS mutants.
