# Supplementary material for: Comparative Epigenomics Reveals that RNA Polymerase II Pausing and Chromatin Domain Organization Control Nematode piRNA Biogenesis
Source: Dev Cell. 2019 Mar 25;48(6):793–810.e6. doi: 10.1016/j.devcel.2018.12.026 (PMC6436959; doi:10.1016/j.devcel.2018.12.026)

Data S2 (relates to Figure 2)

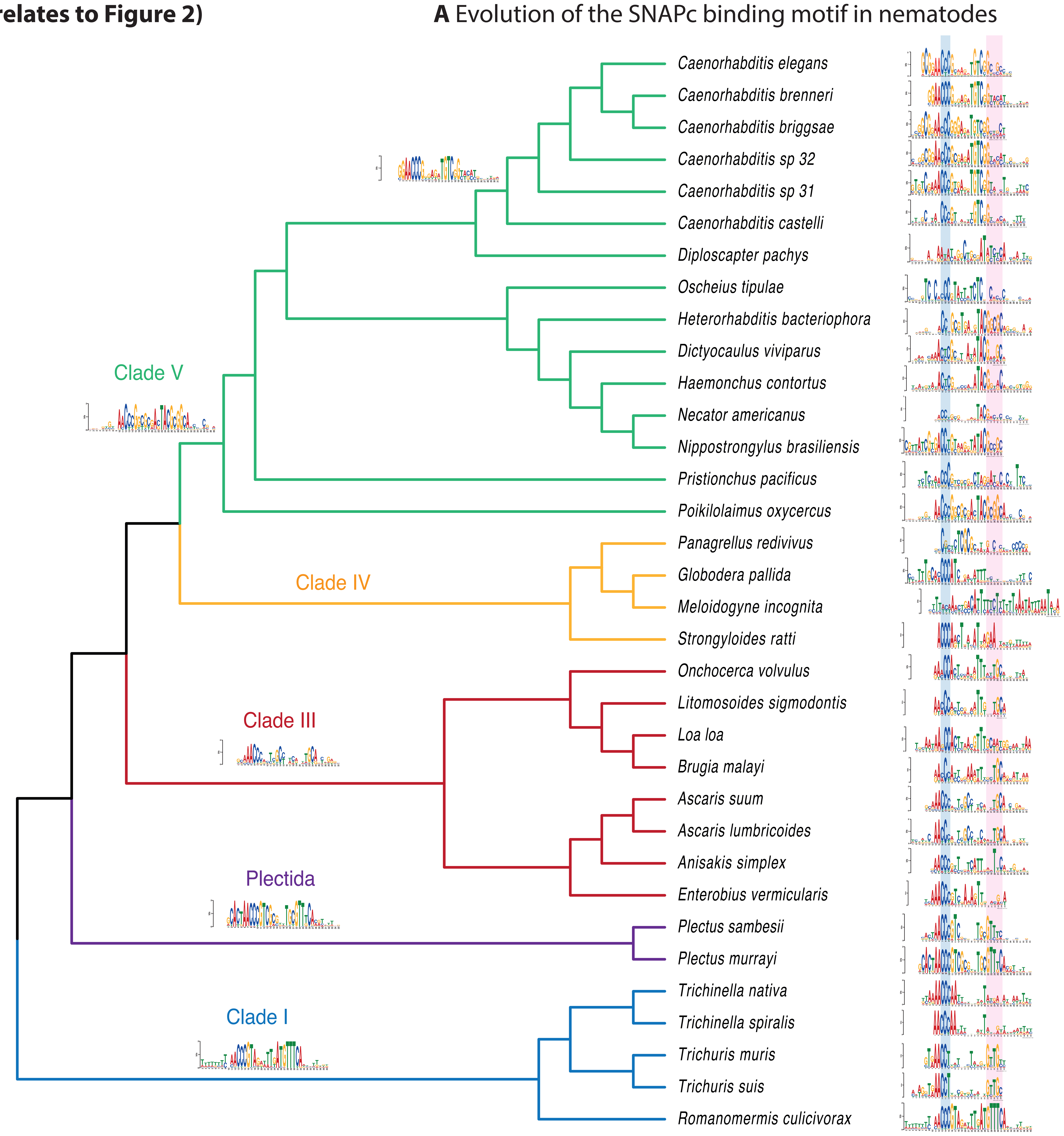

Data S2 (relates to Figure 2)

B Conservation of mammalian SNAPc complex

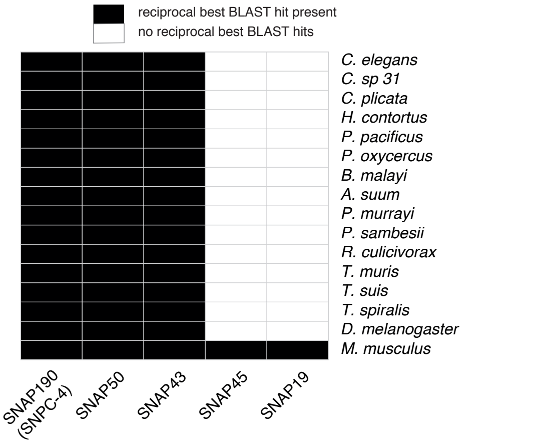

B Phylogenetic tree of nematode SNPC-4 orthologues

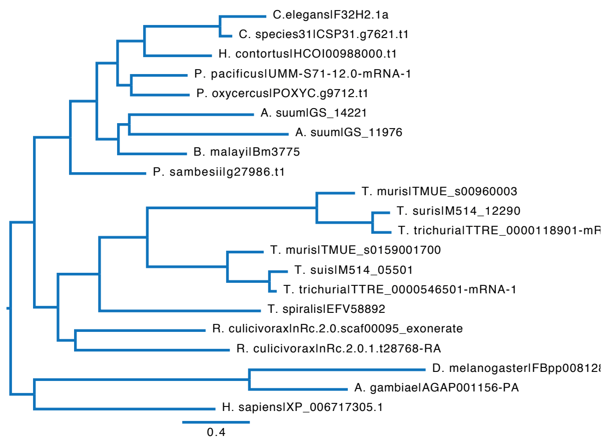

C Overview of the alignment of SNPC-4 orthologs with key regions highlighted

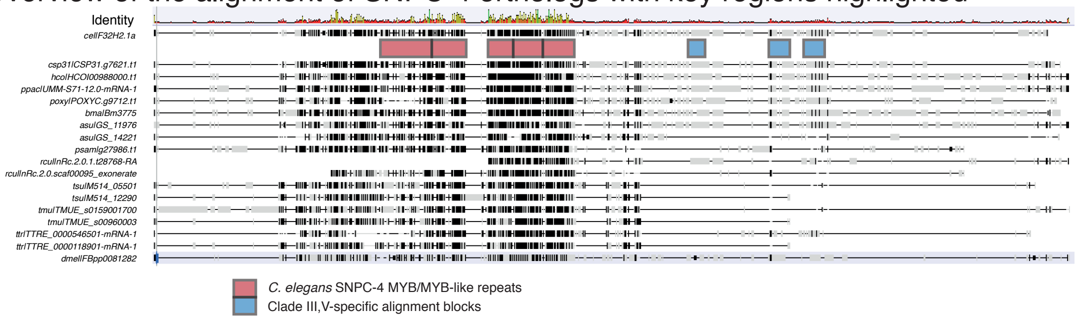

D Alignments of each of the SNPC-4 MYB repeats

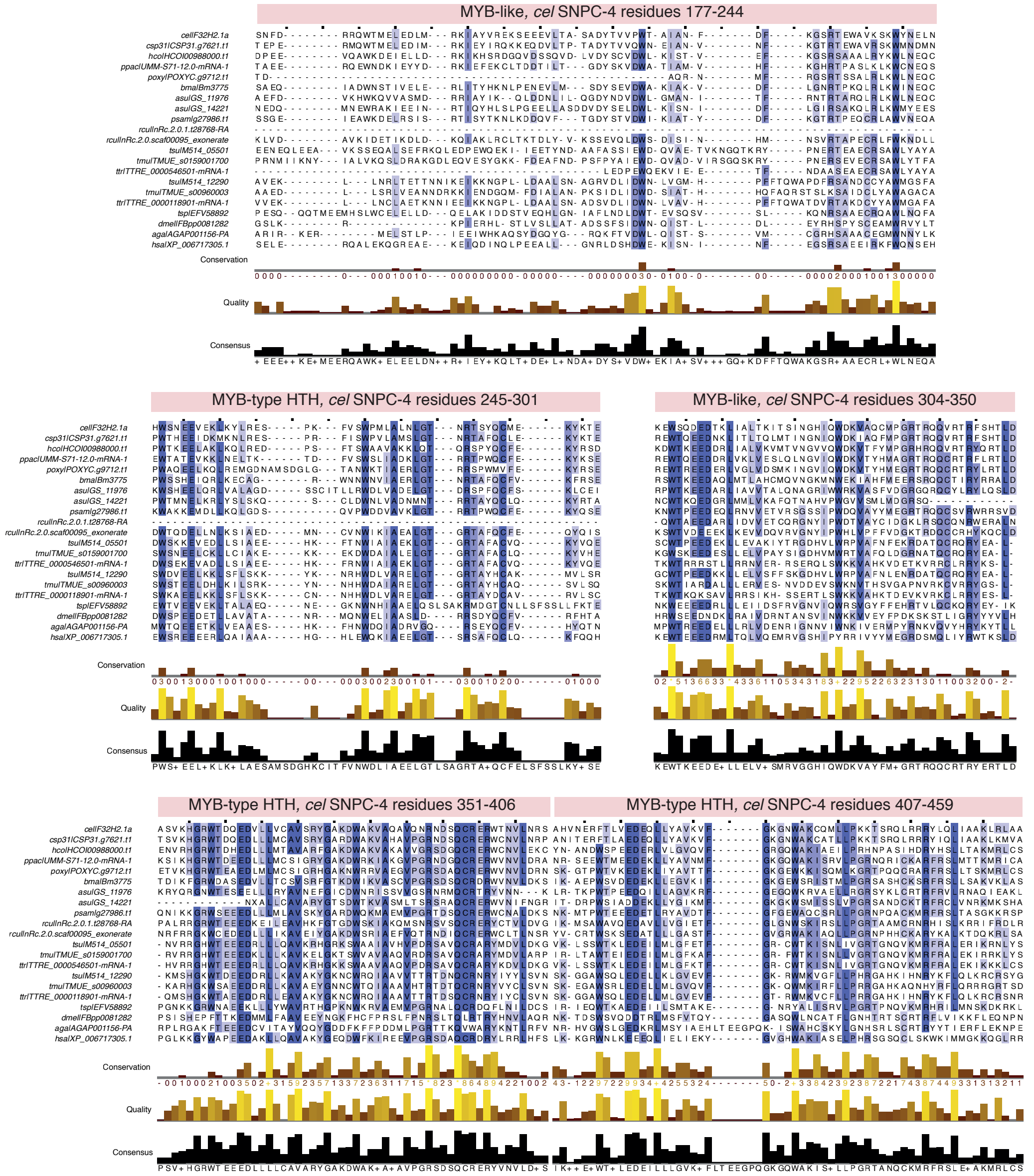

Supplement: Data S2. Evolution of SNPC-4 Orthologs and snRNA Promoter Motifs in Nematodes, Related to Figure 2 — (A) An extended tree with the SNAPc binding motifs across nematodes, along with the predicted ancestral state at key nodes. (B) The presence/absence of human SNAPc complex members in nematodes. (C) A phylogenetic tree of SNPC-4 orthologues across nematodes, with D. melanogaster and H. sapiens SNPC-4 proteins included for comparison. (D) An overview of the alignment of SNPC-4 orthologues with key domains and regions highlighted. D shows alignments of the Myb domains in SNPC-4. [file mmc7.pdf]
